# Awakening antitumour immunity in the multimorbid host: a hypothesis coupling cryoablation-induced immunogenic cell death with multi-target Traditional Chinese Medicine

**DOI:** 10.3389/fimmu.2026.1904534

**Published:** 2026-08-24

**Authors:** Lihui Liu, Honglin Chen, Jingxiao Wang, Jingjing Yang, Yong Zhou, Baoping Luo, Yana Zhou, Kaiwen Hu

**Affiliations:** 1 School of Traditional Chinese Medicine, Hubei University of Chinese Medicine, Wuhan, Hubei, China; 2 Department of Oncology, Hubei Provincial Hospital of Traditional Chinese Medicine, Wuhan, Hubei, China; 3 School of Life Science, Beijing University of Chinese Medicine, Beijing, China; 4 Department of Oncology, Dongfang Hospital, Beijing University of Chinese Medicine, Beijing, China; 5 Hubei Key Laboratory of Theory and Application Research of Liver and Kidney in Traditional Chinese Medicine, Affiliated Hospital of Hubei University of Chinese Medicine, Wuhan, Hubei, China; 6 Hubei Province Academy of Traditional Chinese Medicine, Wuhan, Hubei, China; 7 Hubei Shizhen Laboratory, Wuhan, Hubei, China

**Keywords:** antitumour immunity, cancer multimorbidity, cold-to-hot tumour transformation, cryoablation, immunogenic cell death, immunomodulation, traditional Chinese medicine, tumour microenvironment

## Abstract

Most patients with cancer are older adults living with several other chronic illnesses, and both the tumour and its commonest companions—cardiometabolic disease, type 2 diabetes and depression—are sustained by a shared, immunosuppressive inflammatory milieu. In this setting many tumours are immunologically “cold”: poorly infiltrated by cytotoxic T and natural killer (NK) cells, enriched for regulatory T cells (Tregs) and M2 macrophages, and embedded in a chronically inflamed “soil” that also drives the comorbidities. Here we advance a mechanistic hypothesis: that two low-harm modalities applied together can reawaken antitumour immunity in this host while sparing the physiological reserve that radical treatment consumes. Minimally invasive cryoablation destroys tumour *in situ* and releases tumour antigens together with damage-associated molecular patterns (calreticulin, ATP, HMGB1), driving immunogenic cell death, dendritic-cell maturation and CD8+ T-cell priming; multi-target Traditional Chinese Medicine (TCM) acts on the same inflammatory substrate—dampening NF-κB, IL-6 and TNF-α signalling, lowering lactate, and reducing regulatory-T-cell and M2 dominance—to remodel the tumour microenvironment. We propose that their convergence turns a “cold” microenvironment “hot” and, because the targeted substrate is shared, yields a one-to-many benefit that extends from the tumour to its inflammatory comorbidities. We situate this hypothesis within the three-stage Tumour Green Therapy framework—Dominant Control (*bà dào*, 霸道), Supportive Integration (*wáng dào*, 王道) and Harmonisation and Maintenance (*dì dào*, 帝道)—which sequences local immunogenic debulking, systemic immune support and microenvironmental harmonisation according to tumour urgency and host constitution, and maps TCM syndrome patterns onto measurable immune and inflammatory readouts (neutrophil-to-lymphocyte ratio, CRP, IL-6/TNF-α) that could serve as biomarkers. We define the immunological phenotype of this host, specify which patients should and should not receive the approach, remain candid about its limitations—herb–drug interactions, product quality and the scarcity of randomised data—and pre-specify both a two-stage prospective test and the observations that would refute each element of the hypothesis. Its central, falsifiable claim is that cryoablation plus multi-target TCM reawakens antitumour immunity and reduces total treatment burden, including polypharmacy, without compromising survival or quality of life.

## Background

The global burden of cancer continues to rise. The International Agency for Research on Cancer estimated approximately 20 million new cancer cases and 9.7 million deaths worldwide in 2022 (1). In China, an estimated 4.82 million new cases and 2.57 million cancer deaths occurred in 2022—about one quarter of the world’s cancer mortality—with lung cancer the leading cause of both incidence and death (2). Crucially, advances in early detection, systemic therapy and supportive care have transformed many malignancies from rapidly fatal illnesses into chronic conditions with which patients live for years. As the population of cancer survivors expands and ages, the clinically decisive problem is shifting away from the tumour considered in isolation and toward the patient considered as a whole.

That whole patient is, increasingly, a multimorbid one. In China, cancer mortality is heavily concentrated in older adults: deaths among people aged 60 years and over account for the large majority of all cancer deaths, and a large and growing proportion of patients with cancer are older adults (2), among whom multimorbidity is the norm rather than the exception (3, 4). These patients frequently cannot tolerate radical surgery or full-dose chemoradiotherapy, and conventional, tumour-maximal treatment exposes them to disproportionate harm. The central question for modern oncology is therefore not only how to attack the tumour, but how to construct a gentle, sustainable system of disease control for patients in whom cancer is one of many simultaneous problems.

This review addresses that question. We define cancer multimorbidity and the tools used to measure it; quantify its epidemiological scale and clinical consequences; analyse why the prevailing single-disease treatment model is structurally ill-suited to it; and then present the “Tumour Green Therapy” paradigm developed by Hu and colleagues as a candidate solution. We describe the paradigm’s philosophy and its three-stage operational model, assemble the clinical and mechanistic evidence for its two core modalities—minimally invasive cryoablation and Traditional Chinese Medicine (TCM)—and propose an implementation framework in which artificial intelligence (AI) augments TCM syndrome differentiation. We advance Green Therapy explicitly as a hypothesis-generating paradigm requiring prospective validation, not as an established standard of care. The central, falsifiable hypothesis we develop is this: that minimally invasive cryoablation and multi-target TCM, applied in a stage-adapted sequence, jointly convert immunologically “cold” tumours to “hot” ones and—by acting on the inflammatory substrate shared between cancer and its comorbidities—reawaken antitumour immunity and reduce total treatment burden without compromising survival or quality of life. The sections that follow set out the immunological rationale for this claim, the clinical and mechanistic evidence that bears on it, and how it could be prospectively tested.

### Novelty and relationship to previous work

Three established literatures border on this proposal, and it is worth stating precisely where the present hypothesis departs from each, because it is the combination of elements rather than any single element that is new (**Table 1**).

**Table 1:** Positioning of the present hypothesis relative to adjacent literatures.

Approach	Systemic partner and its target	Intended population	Endpoints in that literature	What the present hypothesis adds
Cryoablation + immune-checkpoint blockade	Monoclonal antibody; one inhibitory receptor (PD-1/PD-L1, CTLA-4) on T cells	Patients fit for immunotherapy; good performance status; few comorbidities	Objective response, progression-free survival, abscopal response	A host-directed rather than synapse-directed systemic arm, tolerable in frail and multimorbid patients for whom checkpoint blockade is closed
Integrative oncology (SIO–ASCO endorsed)	Acupuncture, mind–body and herbal supportive care; symptom pathways	Any patient receiving standard therapy	Pain, anxiety and depression, fatigue	Assigns the traditional-medicine arm a mechanistic role in immune priming, tested against immunological and oncological endpoints
Network pharmacology of compound formulas	Multi-compound, multi-target; predicted in silico	Not population-specific	Predicted target and pathway enrichment; occasional *in vitro* validation	Couples multi-target action to a defined, operator-timed antigen-release event and stratifies by host inflammatory state
Geriatric assessment and deprescribing	Medication review; no antineoplastic mechanism claimed	Older adults with cancer	Treatment toxicity; medications discontinued	Proposes pharmacological substitution by one multi-target agent as the mechanism that makes deprescribing sustainable
Present hypothesis	Multi-target compound formula; host myeloid and inflammatory tone, regulatory T cells, lactate axis	Older, multimorbid, polypharmacy patients ineligible for radical or full-dose therapy, with a dominant ablatable lesion	Immunological conversion and total medication burden, then overall-survival non-inferiority	—

#### Cryoablation combined with immune-checkpoint blockade

That literature couples *in situ* antigen release to pharmacological release of a single inhibitory brake, and has been developed almost exclusively in patients fit enough to receive checkpoint inhibitors (5–7). Its mechanistic target is the T-cell–tumour synapse. The present hypothesis targets a different node—the myeloid and inflammatory tone of the host in which that synapse must form—using an agent class that patients with autoimmune or cardiometabolic comorbidity can plausibly tolerate for months. The two approaches are complementary rather than competing, and we specify below the circumstances in which checkpoint blockade should be preferred.

#### Integrative oncology as currently endorsed

The SIO–ASCO guidelines position Traditional Chinese Medicine–adjacent modalities as supportive care for pain, mood and fatigue alongside standard antineoplastic treatment, and evaluate them against symptom endpoints (8–10). Here the herbal arm is instead assigned a mechanistic role in immune priming and is judged against immunological and oncological endpoints. This is a stronger claim, and correspondingly easier to refute.

#### Network pharmacology of compound formulas

This work generates compound–target–pathway maps in silico (11, 12) but is rarely coupled to a defined immunogenic event *in vivo* and does not stratify by host state. Our hypothesis makes that coupling explicit and time-resolved: the herbal arm is claimed to matter chiefly during and after a discrete, operator-timed antigen-release event.

Against this background the hypothesis reduces to three propositions which, to our knowledge, are not jointly asserted or tested elsewhere.

#### P1 (host-state limitation)

In the multimorbid, chronically inflamed host the rate-limiting barrier to productive *in situ* vaccination is not the availability of tumour antigen but the permissiveness of the host immune compartment—myeloid and regulatory-T-cell tone, senescent T-cell composition, and tonic engagement of damage-sensing pathways.

#### P2 (shared substrate, one-to-many benefit)

Because that substrate is shared with the commonest cancer comorbidities, a single multi-target systemic intervention can produce simultaneous, measurable benefit across tumour and comorbidity, quantifiable as reduced total medication count without loss of comorbidity control.

#### P3 (host state is measurable and actionable)

Syndrome patterns index this substrate closely enough to inform patient selection and the timing of the two arms, and that indexing can be made reproducible by computational means.

Each proposition is paired with a pre-specified refutation condition in **Table 2**.

**Table 2:** Pre-specified falsification criteria for each proposition.

Proposition	Prediction	Observation that would refute it	Where tested
P1 — host-state limitation	Immunological conversion after ablation is greater in patients whose baseline inflammatory tone is reduced by the systemic arm, and greatest in those with the highest baseline tone	Conversion rates equal between arms, or unrelated to baseline IL-6, CRP or neutrophil-to-lymphocyte ratio; or antigen release demonstrated (tier 2c) without downstream antigen-specific expansion in any subgroup	Stage 1 co-primary (i); pre-specified interaction test with baseline IL-6 tertile
P2 — shared substrate, one-to-many benefit	Medication count falls in the experimental arm while objective comorbidity control is maintained or improved	No difference in medication change between arms; or a fall in medication count accompanied by deterioration in HbA1c, blood pressure or depression score, which meets the endpoint in form while refuting the claim in substance	Stage 1 co-primary (ii) with comorbidity-control secondary endpoints
P3 — host state is measurable and actionable	Syndrome assignment correlates with inflammatory and immune measures, and model assistance raises inter-rater reliability	No correlation between syndrome scores and tier 1 or tier 2 measures; or Cohen’s κ unimproved by model assistance; or syndrome assignment adds nothing to prediction of the co-primary endpoints beyond routine variables	Pre-specified correlation and reliability analyses nested in Stage 1
Timing sub-hypothesis	Starting the systemic arm on day 8 yields greater immunological conversion than starting on day 1, because the priming window is preserved	No difference between day 1 and day 8 initiation, or superiority of day 1	Embedded second randomisation, Stage 1
Whole model	Stage-adapted therapy is non-inferior for survival while reducing treatment burden	Inferior overall survival on either intention-to-treat or per-protocol analysis; or non-inferiority achieved with no reduction in treatment burden, which would leave the paradigm without its distinctive claim	Stage 2

### Search strategy

We searched PubMed, Embase, Web of Science, the Cochrane Library and the China National Knowledge Infrastructure for English- and Chinese-language publications, with no lower date limit for foundational concepts and an emphasis on work published between January 2010 and March 2026. Search terms combined keywords for malignancy (“cancer”, “tumour”, “malignancy”) with terms for multimorbidity and comorbidity, polypharmacy, geriatric oncology, cryoablation and argon–helium cryosurgery, integrative oncology, Traditional Chinese Medicine, compound formula, network pharmacology, immunogenic cell death and syndrome differentiation. Reference lists of key articles were screened manually. As a narrative review, this synthesis is interpretive rather than systematic.

## Cancer multimorbidity: definition, measurement and scale

### From comorbidity to multimorbidity

The vocabulary used to describe coexisting illness is not interchangeable, and the distinction matters for how care is organised. Feinstein introduced the term *comorbidity* in 1970 to denote “any distinct additional clinical entity that has existed or that may occur during the clinical course of a patient who has the index disease under study” (13). Comorbidity is therefore an index-disease–centric concept: every other condition is defined relative to one focal disease—in oncology, the cancer. Multimorbidity, by contrast, is person-centric: the co-occurrence of two or more long-term conditions in an individual, without privileging any single one as the index (14). The UK National Institute for Health and Care Excellence (NICE) similarly defines multimorbidity as the presence of two or more long-term health conditions, which may include physical and mental illnesses, ongoing conditions such as frailty or chronic pain, and sensory impairment (15).

The shift from a comorbidity to a multimorbidity perspective is not merely semantic. Comorbidity frames the cancer as central and other conditions as modifiers of cancer treatment; multimorbidity recognises that, for many older patients, no single disease is dominant and that the interactions among conditions—and among their treatments—are themselves the clinical problem. Cancer multimorbidity, the coexistence of cancer with one or more other chronic conditions, thus names a state in which the patient’s overall physiological reserve, treatment tolerance and prognosis are shaped by the entire constellation of disease rather than by the tumour alone.

### Instruments for measuring multimorbidity burden

Several validated instruments quantify comorbidity and multimorbidity burden in oncology, each capturing a different facet of the problem (**Table 3**). They differ chiefly in whether they count weighted diagnoses or grade severity, and in whether they are intended for the bedside or for administrative data: the Charlson Comorbidity Index and its claims-based NCI adaptation weight discrete conditions against mortality (16, 17); the Cumulative Illness Rating Scale and its geriatric modification grade severity across organ systems rather than counting diagnoses, capturing the graded burden characteristic of frail older patients (18, 19); and the Adult Comorbidity Evaluation-27 predicts survival in a dose-dependent, stage-independent manner in cancer-registry populations (20).

**Table 3:** Instruments for measuring comorbidity and multimorbidity burden in oncology.

Instrument	What it measures	Validation in oncology	Strengths	Limitations
Charlson Comorbidity Index (CCI)	Weighted sum of 19 comorbid conditions predicting 1-year mortality	Developed and validated in a breast-cancer cohort (16)	Simple, widely used, strong mortality prediction	Weights pre-date modern therapy; limited within-condition severity gradation; cancer itself is weighted
NCI Comorbidity Index	Charlson conditions adapted to administrative/claims data	Validated in elderly prostate and breast cancer (17)	Enables large registry/claims studies; adding physician claims more than doubles detection	Dependent on coding completeness; not a bedside tool
CIRS/CIRS-G	Severity of illness graded across organ systems	Widely used in geriatric oncology (18, 19)	Captures graded severity across all systems, not mere presence/absence	Requires clinical judgement and training; no single mortality weight
ACE-27	27 conditions across 12 organ systems, graded none–severe	Validated in a hospital cancer registry (20)	Cancer-specific; stage-independent, dose-dependent prognostic value	Requires chart review by trained staff
G8 + comprehensive geriatric assessment	Screening (G8) then multidomain assessment of function, cognition, nutrition, mood, medication	Older cancer patients ≥70 years (21)	Captures functional reserve and treatment tolerance; guides fitness for therapy	G8 alone does not measure comorbidity; full assessment is time-consuming
Polypharmacy (≥5 medications)	Count of concurrent medications	Meta-analysis in older adults with cancer (22)	Simple; both a marker and a modifiable mechanism of harm	Threshold is arbitrary; does not weight appropriateness

Because comorbidity indices do not capture the functional and physiological vulnerability that determines treatment tolerance, geriatric oncology adds dedicated screening and assessment. The G8 screening tool flags older patients who warrant full comprehensive geriatric assessment; at the conventional threshold of ≤14 it achieves a sensitivity of approximately 85% (21). Comprehensive geriatric assessment in turn evaluates function, cognition, nutrition, mood, social support and—critically—medication burden. Polypharmacy, most often defined as the concurrent use of five or more medications, is both a marker and a mechanism of multimorbidity burden and is the single most modifiable element of the problem (22). Together these instruments allow multimorbidity to be staged, much as the tumour itself is staged, and provide the quantitative substrate against which any new management paradigm—including Green Therapy—can be evaluated.

### Epidemiological scale and clinical consequences

**Figure 1:**
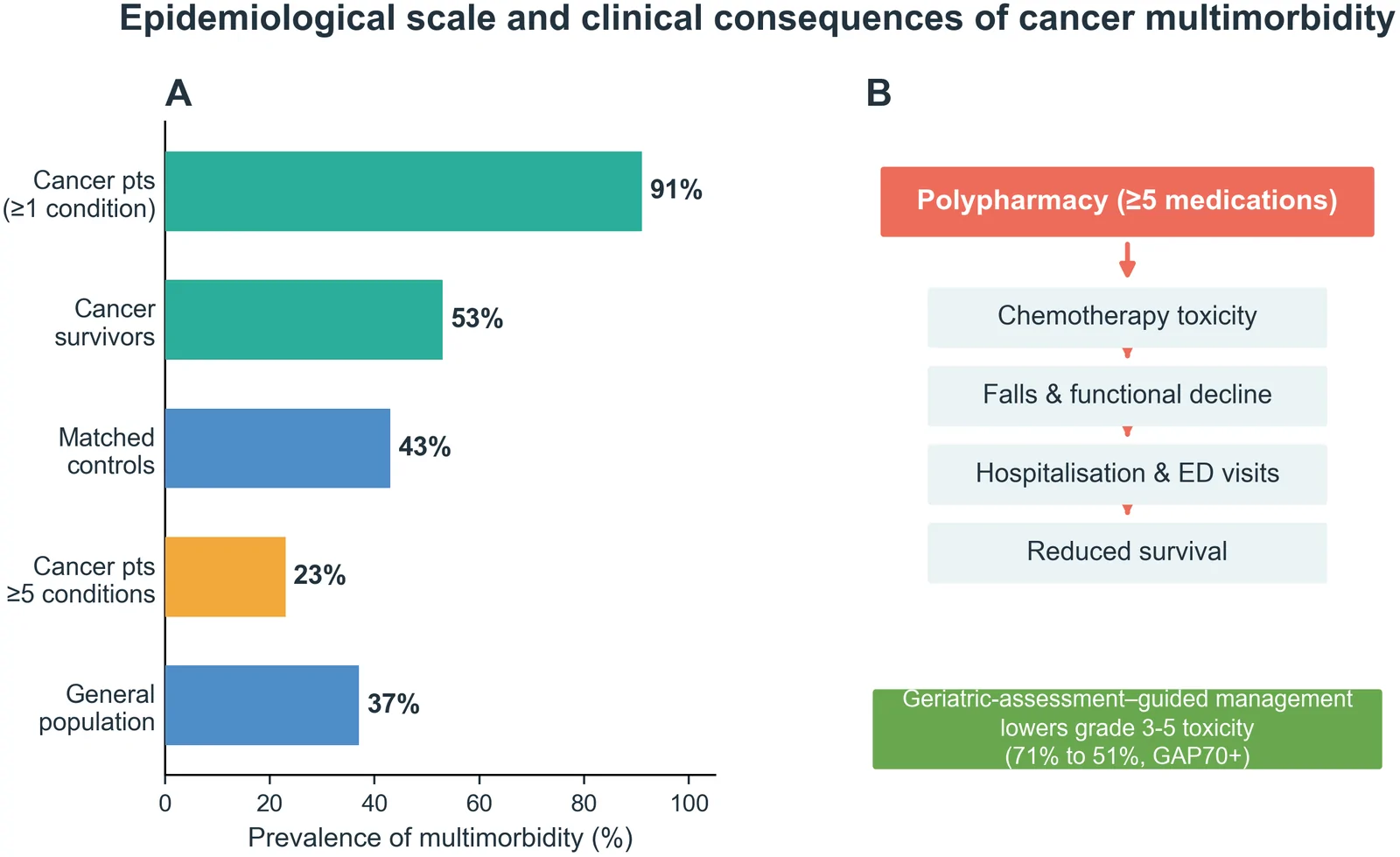
Multimorbidity is highly prevalent among people with cancer, and the resulting polypharmacy drives treatment toxicity, hospitalisation and mortality. Data sources, populations and years: the estimates that more than 91% of adults with cancer have at least one additional chronic condition and that 23% have five or more are from a population-based cohort of 601,331 adults diagnosed with cancer in Ontario, Canada (reported 2021) (3); the comparison of multimorbidity prevalence in cancer survivors (53%) with matched non-cancer controls (43%) is from the Atlantic PATH nested case–control study, Canada (reported 2021) (23); the general-population estimate of approximately 37%, rising above 50% at ages 60 years and over, is a global pooled estimate from a systematic review and meta-analysis of community-dwelling adults (published 2023) (4); and the associations of polypharmacy and potentially inappropriate medications with chemotherapy toxicity, falls, functional decline, hospitalisation and emergency-department visits, postoperative complications and reduced survival are from a meta-analysis of 47 studies in older adults with cancer (published 2020) (22), with the reduction in grade 3–5 toxicity from 71% to 51% under geriatric-assessment-guided care taken from the GAP70+ cluster-randomised trial conducted in the United States (reported 2021) (24). The panel is therefore not a single worldwide estimate: only the general-population prevalence is global, whereas the cancer-specific estimates are region-specific (Canada) and were derived using different definitions of multimorbidity and different ascertainment methods, so they are not directly comparable with one another. **(A)** Prevalence of multimorbidity among cancer patients (91%), cancer survivors (53%), matched non-cancer controls (43%), cancer patients with five or more conditions (23%) and the general population (37%). **(B)** Flowchart linking polypharmacy (≥5 medications) to chemotherapy toxicity, falls and functional decline, hospitalisation and emergency-department visits, and reduced survival, with geriatric-assessment–guided management shown to lower grade 3–5 toxicity from 71% to 51%.

Cancer multimorbidity is common, growing and consequential (**Figure 1**). In a population-based cohort of 601,331 adults diagnosed with cancer in Ontario, Canada, more than 91% had at least one additional chronic condition and 23% had five or more, with multimorbidity rising with age and independently associated with higher health-service utilisation and mortality (3). In the Atlantic PATH nested case-control study, multimorbidity affected 53% of cancer survivors versus 43% of matched non-cancer controls, and survivors took significantly more prescription medications (23)—evidence that cancer accelerates the accumulation of both disease and drugs. At the population level, the global prevalence of multimorbidity in community-dwelling adults is approximately 37%, rising above 50% in those aged 60 and over (4), so the multimorbid older adult is the rule rather than the exception among incident cancer patients.

The downstream harms are measurable. A meta-analysis of 47 studies in older adults with cancer linked polypharmacy and potentially inappropriate medications to chemotherapy toxicity, falls, functional decline, postoperative complications and reduced survival (22). These harms are, importantly, modifiable: in the GAP70+ cluster-randomised trial of 718 adults aged ≥70 with advanced cancer, a geriatric-assessment–guided intervention that included medication review reduced grade 3–5 treatment toxicity from 71% to 51% (relative risk 0.74) and led to more medications being discontinued (24). The implication is that the burden of multimorbidity and its treatment is not an immutable feature of the patient but a tractable target—provided care is organised around the whole person rather than around each disease in isolation.

## Why the single-disease model fails the multimorbid patient

The polypharmacy and treatment burden documented above are not accidental; they are the predictable output of how modern medicine is organised. Subspecialisation has delivered extraordinary gains in diagnostic precision and targeted therapeutics, but each subspecialty generates its own single-condition guideline, and each guideline recommends its own escalating regimen. When these guidelines are summed across a multimorbid patient, the result is an additive prescribing cascade. Boyd and colleagues illustrated this vividly: applying the relevant clinical practice guidelines to a hypothetical 79-year-old woman with five common chronic conditions would prescribe twelve separate medications on a complex daily schedule, with multiple opportunities for drug–drug and drug–disease interaction (25). Single-disease guidelines are, in general, neither derived from nor validated in multimorbid populations, yet they are applied to them by default.

NICE formally acknowledged this structural mismatch in its guideline NG56, which recognised that single-condition guidance is often inappropriate for patients with multimorbidity and called for an explicit shift toward individualised, goal-directed care that reviews treatment burden and optimises or deprescribes medication (15). The diagnosis of structural origin points directly to the therapeutic requirement: what multimorbid cancer patients need is not another subspecialty protocol layered onto the stack, but a framework capable of acting on the shared pathophysiological soil from which several conditions grow at once—a framework in which a single, low-harm intervention can yield benefit across multiple coexisting diseases simultaneously. This is the gap that Green Therapy is designed to fill.

A biological rationale supports the feasibility of such one-to-many intervention. Cancer and its common companions—cardiovascular disease, type 2 diabetes, metabolic syndrome and depression—are not biologically independent; they share converging substrates including chronic low-grade inflammation, oxidative stress and insulin resistance, and dysregulated immune-metabolic signalling (26–28). The epidemiological observation that cancer patients accumulate other chronic conditions faster than age-matched controls reflects this molecular kinship. If multiple coexisting diseases are sustained in part by a common inflammatory and immune-dysregulated milieu, then a therapy that remodels that milieu—rather than one that targets a single receptor in a single organ—offers a mechanistically plausible route to simultaneous, multi-condition benefit. TCM, with its multi-component, multi-target pharmacology and its whole-organism syndrome framework, is a natural candidate for this role.

## Convergent pathological substrates: the biological case for one-to-many intervention

### Four molecular axes link cancer to its commonest companions

The proposition that a single multi-target intervention can benefit several coexisting diseases is credible only if those diseases share modifiable biology. In the multimorbid cancer patient four axes recur with enough mechanistic and clinical definition to serve as rational targets, and—important for the present argument—each maps onto a recognisable Traditional Chinese Medicine syndrome pattern.

First, the inflammation–cachexia axis. Cancer cachexia, a multifactorial loss of skeletal muscle not reversible by nutrition, affects roughly half to four-fifths of patients and may account for up to a fifth of cancer deaths (29, 30). Its proximate driver is interleukin-6 trans-signalling through STAT3, which in murine lung adenocarcinoma is sufficient to produce wasting and is reversed by selective blockade of the soluble IL-6 receptor (31). Clinically this is the fatigued, anorexic, muscle-wasted patient that TCM recognises as spleen–qi deficiency (*pí qì xū*, 脾气虚)—precisely the pattern for which qi-tonifying, fu-zheng formulas built on Astragalus are prescribed.

Second, the inflammation–mood axis. Depression and anxiety affect 30–40% of cancer patients, with major depression in roughly 15% (32), and depression independently predicts higher mortality across 76 prospective cohorts (33). The mechanistic bridge is the same innate-immune activation—IL-6, TNF-α and kynurenine-pathway diversion of tryptophan—that underlies inflammatory depression generally (34). In syndromic terms this is liver–qi stagnation overlaid on qi deficiency, the target of soothing-the-liver (shu gan) strategies.

Third, the insulin–IGF axis. Type 2 diabetes is bidirectionally linked to cancer—an umbrella review found significantly increased risk in 20 of 27 meta-analyses (35)—and metformin use is associated with lower cancer incidence and mortality (pooled relative risk for cancer mortality 0.66) (36), implicating hyperinsulinaemia and IGF-1 signalling as the shared driver (37). This is the phlegm-damp, qi-and-yin-deficient constitution characteristic of metabolic disease.

Fourth, the hypercoagulable axis. Cancer is a prothrombotic state (Trousseau’s syndrome): venous thromboembolism risk is elevated in 18 of 20 cancers, from a hazard ratio of about 1.7 in prostate to 9.7 in pancreatic cancer (38), and is quantified at the bedside by the Khorana score (39). Strikingly, the TCM pattern of blood stasis (*xuè yū*, 血瘀) has a measurable correlate: a meta-analysis of 10,323 patients found blood-stasis syndrome associated with higher platelet aggregation, fibrinogen and plasminogen-activator-inhibitor-1 and shorter activated partial thromboplastin time (40), and the pattern carries a reproducible transcriptomic signature (41).

The clinical significance of this convergence is specific. A compound formula that simultaneously dampens IL-6/NF-κB signalling, improves insulin sensitivity and moderates platelet hyperreactivity is not treating four diseases by coincidence but acting on one shared substrate—exactly the pharmacological profile that network analyses attribute to multi-herb formulas (11, 42). It is this, rather than any vitalist claim, that makes one-to-many intervention biologically plausible.

## Syndrome differentiation as a systems-level readout—and its measurement limits

This mapping reframes syndrome differentiation (bian zheng) not as metaphor but as a coarse, whole-organism readout of the shared substrate. Empirically, syndrome patterns track measurable biology: in untreated colorectal cancer, five TCM syndromes segregate by gut-microbiota diversity and marker taxa (43), and blood stasis maps to coagulation as above. Reliability can be high when the assessment is operationalised—internal consistency of excess/deficiency subgrouping reaches Cronbach’s α 0.87–0.91 (44)—and the predominant constitutional types in lung cancer are reproducibly qi, yang and yin deficiency (45). Intellectual honesty requires the counterweight: tested against practitioner diagnosis, the Constitution in Chinese Medicine Questionnaire shows only fair sensitivity and specificity (42.7–82.7%), and constitutional classification can drift over months (46). Syndrome differentiation is therefore best regarded as a useful but imperfectly standardised instrument—one whose reproducibility is precisely what the artificial-intelligence tools discussed below are intended to improve.

### From multimorbidity to immune dysfunction: the immunological phenotype of the multimorbid host

The four axes above describe shared pathophysiology; they do not yet state what that pathophysiology does to antitumour immunity, or why the intervention proposed here should be particularly suited to it. Because that link is the load-bearing element of the hypothesis, we set it out explicitly before describing the intervention.

### Each common comorbidity converges on a definable immune lesion

Each of the conditions that most often accompanies cancer in older adults maps onto a reasonably well-characterised lesion in the effector arm of antitumour immunity (**Table 4**).

**Table 4:** The immunological phenotype of the multimorbid host: from comorbidity to a predicted consequence for cryoablation-induced *in situ* vaccination.

Comorbidity or host state	Principal immune lesion	Predicted consequence for *in situ* vaccination	Target of the systemic arm	Measure (tier, Table 5)
Type 2 diabetes, hyperglycaemia	Impaired dendritic-cell maturation; glycolytic, poorly phagocytic macrophages; reduced CD8 mitochondrial fitness; tonic RAGE engagement by advanced glycation end-products	DAMP burst sensed less efficiently; higher threshold for dendritic-cell licensing; larger antigen release or adjuvant may be required	Insulin–IGF axis; NF-κB; glycaemic control permitting deprescribing	HbA1c (1); dendritic-cell and CD8 phenotype (2); serum HMGB1 (2)
Obesity, adipose inflammation	Adipose IL-6 and TNF-α; expanded myeloid-derived suppressor cells; leptin-driven PD-1 upregulation and CD8 exhaustion	Exhausted but reinvigoratable T-cell compartment; predicted to benefit more than lean patients, not less	IL-6–STAT3; myeloid suppressor expansion; PD-1/PD-L1 axis	IL-6, CRP (1); PD-1/TIM-3/LAG-3 fractions, suppressor cells (2)
Atherosclerotic cardiovascular disease	NLRP3–IL-1β myeloid activation shared with tumour-promoting inflammation; clonal haematopoiesis sustaining a hyper-inflammatory myeloid compartment	Suppressive myeloid milieu aborts priming; conversely the strongest external precedent for one-to-many anti-inflammatory benefit	NLRP3–IL-1β; NF-κB; macrophage repolarisation	CRP, IL-6 (1); monocyte and macrophage phenotype (2, 3)
Frailty, sarcopenia, immunosenescence	Contracted naive CD8 pool and narrowed T-cell receptor repertoire; accumulation of CD28−CD57+KLRG1+ senescent cells; reduced natural killer cytotoxicity	Attenuated abscopal component; response increasingly dependent on relief of suppression and innate effectors rather than *de novo* priming	Inflammation–cachexia axis; qi-tonifying support of effector reserve	G8, prognostic nutritional index (1); naive/senescent fractions, T-cell receptor repertoire (2)
Chronic low-grade inflammation (inflammaging)	IL-6–STAT3-driven myeloid suppressor expansion; impaired dendritic-cell differentiation; high neutrophil-to-lymphocyte ratio	Established negative predictor of checkpoint-inhibitor benefit; identifies the patients in whom the systemic arm should matter most	IL-6/NF-κB; lactate and HIF-1α; regulatory T cells	Neutrophil-to-lymphocyte ratio, CRP, IL-6 (1); suppressor cells, interferon-γ signature (2, 3)
Depression, chronic stress	β-adrenergic and glucocorticoid suppression of T-cell effector function and dendritic-cell trafficking	Reduced priming efficiency and effector trafficking independent of tumour biology	Inflammation–mood axis; liver-qi-soothing strategies	Depression score, IL-6 (1); effector trafficking markers (2)

#### Type 2 diabetes and hyperglycaemia

*In vivo*, sustained hyperglycaemia impairs dendritic-cell maturation and antigen presentation, shifts macrophages towards a glycolytic but poorly phagocytic phenotype, and constrains the mitochondrial fitness on which CD8^+^ effector and memory differentiation depends; regulatory T-cell frequency rises and Th1 polarisation falls (47, 48). We specify *in vivo* deliberately: the direction of the dendritic-cell effect is model-dependent, and some high-glucose work *in vitro* reports enhanced rather than impaired maturation, so we restrict the claim to the tumour-bearing setting with which this hypothesis is concerned (47). Advanced glycation end-products chronically engage RAGE, which is also a principal receptor for HMGB1; in the diabetic host, therefore, the damage-sensing system is already chronically active rather than quiescent, sustaining a sterile, myeloid-dominated inflammation (49). This carries a consequence for the present hypothesis: the damage-associated molecular pattern (DAMP) burst generated by ablation is superimposed on a high background, so its incremental signal is proportionally smaller, and it arrives in a milieu whose existing inflammation is not productive for antigen-specific priming. The diabetic host may therefore require a larger antigen and DAMP release, or explicit adjuvant support, to cross the threshold for dendritic-cell licensing. We note that this is an argument from signal-to-background and from inflammatory context, not from receptor desensitisation, for which we are not aware of direct evidence.

#### Obesity and adipose inflammation

Adipose tissue sustains IL-6 and TNF-α production, expands myeloid-derived suppressor cells, and—through leptin signalling—drives PD-1 upregulation and functional exhaustion of CD8^+^ T cells (50). A body of clinical evidence then reports an “obesity paradox”: improved progression-free and overall survival on checkpoint blockade at higher body-mass index (51, 52). That literature is contested and should be reported as such. The melanoma association was driven mainly by male patients; subsequent cohorts have not consistently reproduced elevated PD-1 in obese patients or animals; and at least one analysis restricts the effect to overweight and class-I obesity and suggests that skeletal muscle mass rather than adiposity is the operative variable (53, 54). We regard this uncertainty as useful rather than damaging, because it converts an assumption into a discriminating test: if the obese host’s T-cell compartment is exhausted but reinvigoratable, supplying antigen while lowering myeloid and inflammatory tone should benefit these patients *more*, not less, than lean ones; whereas if adiposity is merely a proxy for preserved muscle mass, any effect should track the frailty and sarcopenia measures of **Table 5** instead. The two predictions are distinguishable within the design proposed below, and the naive expectation that comorbidity uniformly predicts failure is refuted either way.

**Table 5:** Proposed layered biomarker strategy, with sampling schedule and the claim each tier tests. Day 0 is the day of cryoablation.

Tier	Assays	Sampling schedule	Claim tested	Feasibility
1 — feasibility (all sites, all patients)	Full blood count with neutrophil-to-lymphocyte and lymphocyte-to-monocyte ratios; CRP; albumin and prognostic nutritional index; IL-6; TNF-α; lactate dehydrogenase; HbA1c; lipids; blood pressure; depression score; Charlson index, G8, medication count, STOPP/START flags	Baseline; days 1, 3, 7; weeks 2, 4, 8, 12; then every 12 weeks	P2 (one-to-many): inflammatory tone falls while comorbidity control is maintained and medication count falls	Routine; county-level hospital
2a — immune phenotype (central laboratory)	Flow cytometry: CD8/regulatory T-cell ratio; naive, memory and effector distribution; senescent (CD28−CD57+KLRG1+) and exhausted (PD-1+TIM-3+LAG-3+) fractions; monocytic and granulocytic myeloid-derived suppressor cells; natural killer frequency with NKG2D and perforin	Baseline; day 7; weeks 4, 12	P1 (host-state limitation): suppressive tone falls and effector fraction rises	Central laboratory; requires fresh-sample logistics
2b — antigen-specific function	Interferon-γ ELISpot against autologous tumour lysate; interferon-γ release on polyclonal stimulation	Baseline; weeks 4, 12	P1: the most direct available test of *in situ* vaccination	Demanding; requires banked autologous lysate. Priority for funding
2c — DAMP and interferon burst	Serum HMGB1; soluble calreticulin; cell-free DNA; type I interferon gene signature	Baseline; hours 6, 24, 72	Step 1–2 of the cascade actually occurred in this host	Feasible; strict pre-analytical timing
2d — molecular response and clonality	Circulating tumour DNA (variant allele fraction, molecular response); T-cell receptor β repertoire sequencing (clonality, diversity)	Baseline; weeks 4, 12; then every 12 weeks	P1 and efficacy, tumour-agnostic, where post-ablation imaging is uninterpretable	Available commercially; cost is the constraint
3 — tissue (pre-specified subgroup)	Paired biopsy of a distant UNABLATED index lesion: CD8+ and CD103+ density, PD-L1 (tumour proportion and combined positive score), CD68/CD163 macrophage phenotype, Foxp3+ regulatory T cells, interferon-γ-related gene-expression signature; baseline tumour mutational burden and microsatellite status	Baseline and week 4	P1, and excludes the alternative that benefit reflects a conventionally immunogenic tumour rather than a modified host	Only where a safely accessible second lesion exists; target 40–50% of participants

#### Atherosclerotic cardiovascular disease

Atherosclerosis and tumour-promoting inflammation share a myeloid driver in the NLRP3–IL-1β axis, and the clearest existing proof of principle for one-to-many anti-inflammatory intervention comes from this axis: in a large secondary-prevention trial of interleukin-1β blockade, cardiovascular event rates and lung-cancer incidence and mortality fell together (55, 56). Clonal haematopoiesis of indeterminate potential, prevalent in the same age group, links cardiovascular risk to a constitutively hyper-inflammatory myeloid compartment and plausibly to impaired antitumour immunity (57). This is the single strongest external support for P2, and it was achieved with a monoclonal antibody against one cytokine; the claim advanced here is that a multi-target agent acting on several nodes of the same network could achieve a comparable one-to-many effect at a fraction of the cost.

#### Frailty, sarcopenia and immunosenescence

The naive CD8^+^ pool contracts steeply from approximately 65 to 70 years of age—the lower age bound of the population this hypothesis concerns—and T-cell receptor repertoire diversity narrows, while senescent CD28^−^CD57^+^KLRG1^+^ T cells accumulate with poor proliferative capacity and natural killer cytotoxicity declines with reduced NKG2D and perforin expression (58–60). Whether the loss of diversity is driven principally by reduced thymic output or by peripheral homeostatic proliferation remains debated, and the distinction matters here in a direction that favours caution rather than optimism: homeostatic proliferation preserves cell number without generating new receptors, so on that account the naive repertoire actually available to recognise ablation-released antigen is more restricted than a cell count would imply. This matters disproportionately here, because *in situ* vaccination requires the recruitment of previously unengaged naive clones. The hypothesis therefore predicts that the abscopal component of the response will attenuate as frailty increases, and that in the frailest patients any benefit will depend more on relief of suppression and on innate effectors than on *de novo* priming—which is why naive T-cell frequency and repertoire diversity are stratification variables rather than exploratory ones in the design proposed below.

#### Chronic low-grade inflammation as the common final path

Elevated IL-6 and C-reactive protein drive STAT3-dependent expansion of myeloid-derived suppressor cells and impair dendritic-cell differentiation, and a high baseline neutrophil-to-lymphocyte ratio and CRP are repeatedly associated with failure of checkpoint blockade across tumour types (61, 62). This is an important convergence for the argument: the inexpensive markers this paradigm proposes to follow are not merely convenient proxies for a syndromic construct but established negative predictors of immunotherapy benefit, which supplies the mechanistic reason for expecting that lowering them should matter.

#### Depression and chronic stress

Sustained β-adrenergic and glucocorticoid signalling suppresses T-cell effector function and dendritic-cell trafficking, and provides the immunological bridge between the inflammation–mood axis described above and tumour control (63–65). One result in this literature bears directly on the present proposal: β-adrenergic blockade improves CD8^+^ T-cell *priming* and the efficacy of cancer vaccination, acting on the priming phase rather than on established effectors (63–65). If chronic stress falls preferentially on priming, it constrains precisely the step on which cryoablation-induced *in situ* vaccination depends, and it identifies depression in this population as a potentially modifiable determinant of response rather than a comorbidity to be managed separately.

### Why this host, and not another, should benefit

Three consequences follow, and they are what make the hypothesis specific rather than merely plausible.

First, the multimorbid host is antigen-poor in a functional but not an absolute sense: tumour antigen exists and is not being presented, because dendritic-cell maturation is impaired and damage sensing is desensitised. An operator-controlled necrotic event that releases a large bolus of autologous antigen together with calreticulin, ATP and HMGB1 is therefore a rational way to force the first step of a cascade that this host will not take spontaneously—and it is one of very few interventions that supplies antigen without imposing the systemic immunosuppression that radical surgery and full-dose cytotoxic therapy impose.

Second, forcing antigen release is insufficient on its own in precisely this host, because the downstream steps are the broken ones. High myeloid and regulatory tone, an IL-6/STAT3-dominated milieu, lactate-rich microenvironments and a contracted naive T-cell pool will abort priming even when antigen is abundant. This supplies a mechanistic reading of an otherwise awkward clinical result: indiscriminate cryoablation after chemotherapy failure improved disease control and quality of life without prolonging survival (66), which on this account is what should happen when a local immunogenic event is delivered into a host unable to convert it into systemic immunity. The systemic arm is therefore a necessary condition rather than an adjunct, and the hypothesis predicts that its benefit will be greatest in patients with the highest baseline inflammatory tone—a prediction that is directly testable and, if false, fatal to P1.

Third, because the same inflammatory tone sustains the comorbidities, the systemic arm has a second and logically independent output: comorbidity control, and the deprescribing it permits. This is the one-to-many claim, and it is measurable in the same patients at the same visits as the immunological claim, which is what allows the two to be tested together rather than in separate literatures.

## Tumour green therapy: a low-harm paradigm for the multimorbid patient

### Origin and philosophy

Confronting the reality that a large proportion of patients present at intermediate or advanced stages, or are too old, frail or multimorbid to tolerate radical treatment, Hu and colleagues proposed the concept of “Tumour Green Therapy” (*zhǒngliú lǜsè zhìliáo*, 肿瘤绿色治疗) in 2004 and pioneered a “minimally invasive plus TCM” model of care (67, 68). Green Therapy reframes the objective of cancer care: rather than pursuing maximal tumour eradication at any physiological cost, it seeks sustainable, low-harm *control* of the disease, with prolonged survival and preserved quality of life as the primary endpoints (69). It is, in the authors’ framing, a “Chinese solution” that integrates classical Chinese medical philosophy with contemporary minimally invasive technology, and it has since broadened from treatment into a “green prevention-and-treatment” system spanning the disease course. We note our position explicitly: several of the present authors proposed and have since developed this paradigm, and this article is therefore intended not as a neutral systematic appraisal but as a framework and research agenda, in which the supporting evidence is presented alongside its limitations and weighed by the reader accordingly.

Two classical principles anchor the philosophy. The first is *control rather than radical cure*. The *Su Wen* (Liù Yuán Zhèng Jì Dà Lùn) counsels that, in treating great accumulations, one should “attack but stop when more than half is gone; going beyond this leads to death,” and the modern recognition that micrometastasis often occurs before a tumour reaches one centimetre underscores that complete eradication is frequently an unattainable ideal (68). Green Therapy therefore explicitly accepts “living with tumour” (*dài liú shēng cún*, 带瘤生存) as a legitimate and often optimal goal. The second is the *theory of the protective field* (*hù chǎng*, 护场), drawn from the surgical classic *Zheng Zhi Zhun Sheng*: the rim of reactive tissue surrounding a lesion represents the patient’s marshalled vital qi (zheng qi) forming a barrier that constrains the pathogen. During local treatment this protective field should be preserved rather than obliterated—an injunction that aligns with the principle of debulking the tumour core while sparing the host, and with the observation that subclonal populations at the tumour periphery may differ biologically from the core (68). Both principles converge on a single clinical stance: strike the pathogen decisively but proportionately, and never at the expense of the host’s own defensive capacity.

### The three-stage model and its two core modalities

**Figure 2:**
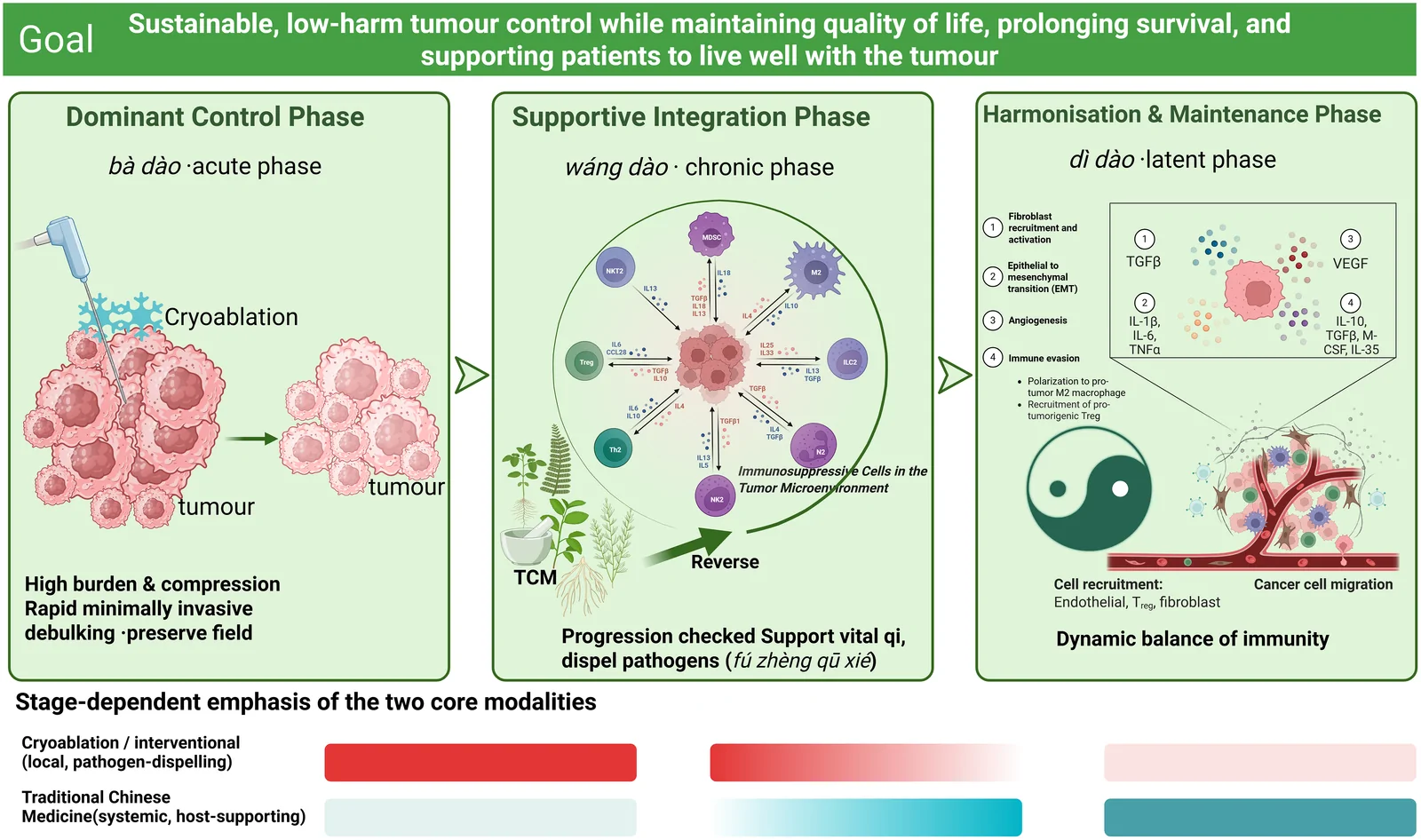
The three-stage green therapy model (dominant control/supportive integration/harmonisation and maintenance phases) and its two core modalities — minimally invasive cryoablation and Traditional Chinese Medicine — deployed singly or in combination by stage. Created in BioRender. Li, L. (2026) https://BioRender.com/hub9ep4.

Green Therapy operationalises this philosophy through a three-stage model that, unlike conventional anatomical staging, is defined by the urgency of the tumour and the constitution of the patient. Each stage takes a functional name that renders a classical mode of governance from Chinese statecraft—Dominant Control (*bà dào*, 霸道), Supportive Integration (*wáng dào*, 王道) and Harmonisation and Maintenance (*dì dào*, 帝道)—terms used here in their classical sense, as ascending modes of rule (by force, by virtue and by effortless harmony, respectively) and not in any modern geopolitical sense—each stage emphasising a distinct therapeutic principle (68): the Dominant Control phase directly targets the cancer focus (Elimination, xiāo); the Supportive Integration phase supports vital qi and dispels pathogens (Tonification, bǔ); and the Harmonisation and Maintenance phase improves the internal environment (the method of harmonisation, hé) (**Figure 2**):

#### Dominant control phase (acute phase)

When tumour burden is high and local compression symptoms are severe, treatment leads with rapid debulking. Minimally invasive local therapy—chiefly cryoablation, and where appropriate interventional/transarterial techniques, thermal ablation or photodynamic therapy—or TCM external treatment serves as the vanguard, with the method of “elimination” (*xiāo*, 消) dominant. The aim is to reduce local tumour burden quickly, relieve compressive symptoms and reverse the momentum of the disease, converting an acute crisis into a controllable chronic state while preserving the protective field.

#### Supportive integration phase (chronic phase)

Once progression is checked—typically in the peri-procedural period—management turns to systemic syndrome differentiation and the TCM strategy of “supporting vital qi and dispelling pathogens” (*fú zhèng qū xié*, 扶正祛邪), with the method of “tonification” (*bǔ*, 补) dominant. Here TCM compound formulas, and where indicated low-dose chemoradiotherapy or biological agents, are used to shift the balance of power between pathogen and host and to reawaken the body’s own antitumour capacity.

#### Harmonisation and maintenance phase (latent phase)

When pathogen and vital qi reach relative equilibrium, the governing principle becomes “harmonisation” (*hé*, 和): improving the internal milieu so that it is unfavourable to tumour growth, correcting the underlying “cancer constitution,” restoring yin–yang balance and preventing recurrence. The ultimate goal is a patient who is both long-lived and well—what the tradition calls the way of “fortune and longevity together.”

A defining feature of this model is that Western and Chinese modalities are not applied as a rigid simultaneous stack but *sequentially* or in a *primary/secondary* relationship that shifts with stage (68). The system is built on two core, low-harm modalities: (i) minimally invasive cryoablation/interventional therapy as the local, pathogen-dispelling arm, and (ii) Traditional Chinese Medicine as the systemic, host-supporting arm. Critically, these may be deployed *alone or in combination* depending on stage and patient: cryoablation alone for a localised, compressive lesion in a frail patient; TCM alone for a patient unfit for or declining any invasive procedure, or in long-term maintenance; or the two combined when local debulking and systemic regulation are both required. This flexibility is precisely what a multimorbid population requires, because it allows the intensity and invasiveness of treatment to be titrated to a patient’s reserve rather than dictated by a disease-specific protocol. Crucially, the two arms map onto the two faces of the multimorbid patient. The local arm does not itself treat the coexisting illnesses; its contribution is to achieve tumour control at a fraction of the physiological cost of radical surgery or full-dose chemoradiotherapy, sparing the functional reserve and immune competence that a frail, multimorbid patient cannot afford to lose. The systemic arm carries the burden of multimorbidity proper: by acting on the shared inflammatory, insulin–IGF and procoagulant substrate set out below, a single multi-target formula can benefit the tumour and several of its comorbidities simultaneously, making rational deprescribing—rather than the additive prescribing cascade of single-disease care—achievable. Tumour control and reduction of whole-patient treatment burden are thus pursued in parallel rather than traded off against each other.

The contrast with radical surgery is instructive (**Table 6**). Compared with open resection, minimally invasive ablation entails a small wound, light tissue injury, rapid operation, minimal bleeding and local rather than general anaesthesia; it debulks locally while sparing immune function and can convert killed tumour cells into a source of antigen rather than discarding them (70, 71). These features make ablation particularly suitable for complex tumours and for elderly, frail patients with limited physiological reserve—exactly the multimorbid population that radical surgery serves least well.

**Table 6:** Radical surgery versus minimally invasive cryoablation in the multimorbid patient.

Feature	Radical surgery	Minimally invasive cryoablation
Wound and tissue injury	Large incision; extensive injury	Percutaneous puncture; limited injury
Operative time	Long	Short
Bleeding	Often substantial	Minimal
Anaesthesia	Usually general	Local (± sedation)
Therapeutic goal	Complete eradication	Local debulking and disease control
Effect on host immunity	Marked perioperative suppression	Relatively preserved; releases tumour antigens (*in situ* vaccine effect)
Complications and recovery	More frequent; slower recovery	Fewer; faster recovery
Best-suited patients	Fit patients; large tumours requiring full clearance	Elderly, frail, multimorbid patients; complex or unresectable tumours

## Clinical evidence I: cryoablation combined with Traditional Chinese Medicine

The local arm of Green Therapy rests on cryoablation, in which an argon–helium or composite cryo-thermal probe is placed percutaneously into the tumour under image guidance to destroy it by freezing. In TCM terms, the rapidly proliferating, metabolically active tumour is a manifestation of “excess heat,” and cryoablation embodies the classical treatment principle “treat heat with cold” (*rè zhě hán zhī*, 热者寒之); the procedure debulks the lesion while, by design, preserving the surrounding protective field (68).

Clinical experience in the target population is encouraging, although the controlled evidence remains early. In a retrospective study of 119 patients with primary lung cancer—most of them elderly or with advanced disease—percutaneous cryoablation combined with TCM was associated with a median overall survival of 19 months from diagnosis, with comparable outcomes whether or not classic anticancer therapy was added, supporting cryoablation-plus-TCM as a viable principal therapy for patients who cannot tolerate standard treatment and emphasising its favourable quality-of-life profile (72). Randomised work is now maturing. A multicentre, non-inferiority randomised controlled trial in stage III–IV non-small cell lung cancer found composite cryo-thermal (“co-ablation”) to be non-inferior to argon–helium cryoablation, with high disease-control rates and comparable complication rates in both arms (73). A multicentre randomised trial protocol formally evaluating Green Therapy—cryoablation plus a 28-day TCM regimen versus platinum-doublet chemotherapy—in stage IIIb/IV non-small cell lung cancer has been registered, with overall survival as the primary endpoint (74). Tempering these results, a separate multicentre randomised trial of cryoablation after first-line chemotherapy failure in advanced non-small cell lung cancer found no overall-survival benefit from indiscriminate cryoablation (median 9.0 versus 11.2 months), although disease control and quality of life favoured the cryoablation arm, indicating that patient selection is decisive and that cryoablation is best understood as a tool for local control and symptom relief within an integrative strategy rather than as a universal life-prolonging intervention (66). Taken together, the current clinical evidence is sufficient to establish cryoablation combined with TCM as a feasible, low-harm option for elderly and multimorbid patients with localised symptomatic disease, but not yet to confirm a survival advantage; adequately powered pragmatic trials with pre-specified survival, quality-of-life and treatment-burden endpoints are required.

A mechanistic caveat tempers enthusiasm and carries direct procedural implications. Cryoablation is immunologically double-edged: depending on freezing parameters it can prime a Th1, tumour-specific response or, conversely, expand regulatory T cells and worsen metastasis. In the 4T1 model a rapid freeze increased tumour-specific T cells and reduced pulmonary metastases, whereas a slow freeze raised regulatory T cells and significantly *increased* metastases and mortality (75, 76). This is not a peripheral detail—it means the immunostimulatory benefit that Green Therapy invokes is contingent on technique, and it supplies a mechanistic rationale for the present group’s own optimisation of freeze–thaw parameters and “non-extended” cryoablation (77). It also argues for coupling ablation with immune support rather than relying on it alone, consistent both with early clinical work combining cryoablation with checkpoint blockade in breast cancer (5–7) and with the fu-zheng herbal arm of the integrative model. The abscopal regression of distant disease after local ablation, although real, remains inconsistent and insufficient as a monotherapy (78)—which is precisely why Green Therapy pairs local debulking with systemic regulation rather than treating ablation as curative in itself.

## Clinical and mechanistic evidence II: TCM as multi-target therapy that awakens immunity

### The pharmacological logic of multi-target therapy

The systemic arm of Green Therapy exploits a pharmacological logic opposite to that of conventional drug design. Western pharmaceuticals are typically engineered to engage a single molecular target with high affinity, an approach that is powerful against a single disease but that, summed across a multimorbid patient, drives the additive prescribing cascade. TCM compound formulas instead deliver many bioactive constituents that act on many targets across several pathways at once. Network-pharmacology analyses make this concrete: a single multi-herb formula can contain scores of bioactive compounds engaging more than a hundred disease targets enriched across dozens of pathways, including the PI3K–Akt, NF-κB, HIF-1 and VEGF signalling axes that are common to cancer and its cardiometabolic companions (11, 12). Work from the present group illustrates the approach: integrated network pharmacology with experimental verification dissected how the *Hedysarum Multijugum* (Astragalus)–*Curcumae Rhizoma* herb pair acts through multiple compounds and targets against non-small cell lung cancer (42). Because these are the very pathways that link cancer to cardiovascular, metabolic and mood comorbidity (26, 27), a multi-target agent that modulates several of them simultaneously is mechanistically suited to one-to-many benefit. Curcumin provides a well-characterised exemplar of a single botanical agent acting on shared nodes—NF-κB, STAT3, COX-2 and reactive oxygen species—across cancer, diabetes, obesity and cardiovascular and neurological disease (79, 80). Comparable multi-target, microenvironment-modulating actions have been reported by independent groups for diverse agents and formulas, from ginseng in colorectal cancer to bezoar-containing preparations in hepatocarcinoma, each acting through multiple components, targets and pathways to regulate the tumour immune microenvironment (81, 82).

## Experimental evidence for direct immune regulation, and what it does not yet show

The evidence adduced so far for immunomodulation by TCM rests substantially on reviews and on network-pharmacology predictions, which are hypothesis-generating rather than confirmatory. We therefore separate deliberately the claims that rest on direct experimental demonstration from those that do not. Several constituents have been shown in cell and animal experiments to act on the specific nodes that the present hypothesis requires: polysaccharide fractions of *Astragalus membranaceus* and of medicinal fungi upregulate CD80/CD86 and MHC class II on dendritic cells and enhance cross-presentation, in part through TLR4-dependent signalling (83, 84); baicalin and curcumin repolarise tumour-associated macrophages from an M2 towards an M1 phenotype in tumour-bearing animals, with suppression of M2-associated STAT3 and arginase-1 and induction of M1-associated STAT1, inducible nitric oxide synthase and type I interferon signalling (85, 86). The direction of such effects is, however, context-dependent: constituents that favour M1 polarisation within a tumour have been reported to favour M2 polarisation in atherosclerotic lesions (87), a caveat of direct relevance to the multimorbid patients this hypothesis concerns. several constituents reduce regulatory T-cell and myeloid-derived suppressor-cell frequencies in tumour-bearing mice (88); and individual compounds modulate tumour PD-L1 expression and restore effector T-cell and natural killer cell function, in part through the lactate and glycolytic reprogramming we have reviewed elsewhere (89–91).

We are explicit about the limits of this evidence, because they bound what it can support. It is predominantly murine; it uses purified single constituents at concentrations that may not be attained by oral decoctions or granules; it seldom demonstrates that a whole compound formula reproduces the effect of its isolated constituents; and it is almost never performed in the aged or metabolically abnormal hosts that this hypothesis concerns. Establishing that a standardised compound formula reproduces these effects at clinically attainable exposures in aged, diet-induced-obese or diabetic tumour-bearing animals is, in our view, the single most important preclinical gap, and we list it as a priority below.

## Pivotal clinical evidence for compound formulas

The strongest clinical evidence that TCM compound formulas are genuine therapeutic agents comes from large randomised trials. In a multicentre, randomised, controlled phase IV trial, the Huaier granule (an aqueous extract of *Trametes robiniophila* Murr) given as adjuvant therapy after curative resection of hepatocellular carcinoma in 1,044 patients across 39 Chinese centres prolonged recurrence-free survival (hazard ratio 0.67, 95% CI 0.55–0.81) and improved the overall-survival rate (95.19% versus 91.46%) while reducing extrahepatic recurrence (92). PHY906, a standardised four-herb formula derived from the Huangqin decoction, reduced chemotherapy- and radiation-induced gastrointestinal toxicity in early-phase trials without altering the pharmacokinetics of the cytotoxic agents, acting simultaneously through promotion of intestinal stem-cell regeneration, anti-inflammatory inhibition of NF-κB, COX-2 and iNOS, and restoration of the gut epithelium—a single formula engaging multiple mechanisms at once (93–95). Adjuvant injections such as Shenqi Fuzheng, evaluated in meta-analyses of dozens of randomised trials in non-small cell lung cancer, are associated with improved response and quality of life and reduced chemotherapy toxicity, although the authors of those syntheses caution that the quality of the primary studies is variable (96, 97). Mechanistic reviews of individual herbs commonly prescribed in oncology—such as *Astragalus membranaceus*, with its immunomodulatory and gastrointestinal-protective actions—provide the constituent-level rationale for these formula effects (98).

## Awakening immunity: ablation and herbs as partners

**Figure 3:**
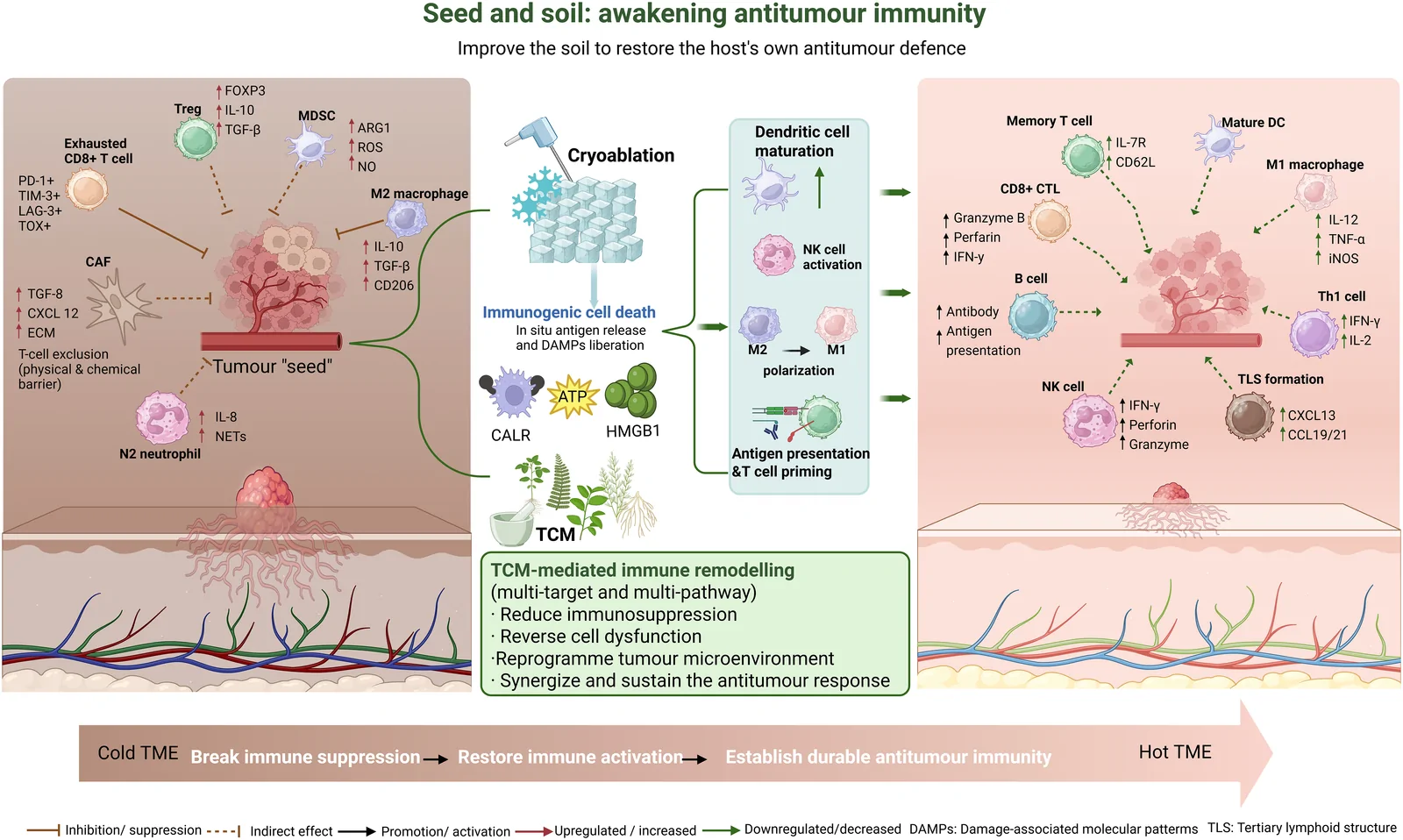
“Seed and soil”: *in situ* cryoablation (antigen release) and multi-target TCM (immune-microenvironment remodelling) cooperate to turn “poor soil” into “good soil” and restore antitumour immunity. Created in BioRender. Li, L. (2026) https://BioRender.com/5rkjya3.

The deepest synergy between Green Therapy’s two modalities lies in immunity. In TCM image-thinking, the tumour is a “seed” and the host milieu its “soil”; the therapeutic priority is less to eliminate every seed than to improve the soil—to restore the patient’s own defensive capacity (**Figure 3**). Modern immunology supplies a mechanistic translation of this metaphor. Cryoablation does not merely destroy tumour cells; by killing them *in situ* it releases a full repertoire of autologous tumour antigens together with danger-associated molecular patterns, providing the raw material for an *in situ* tumour vaccine and modulating the tumour immune microenvironment, including regulatory T-cell and TGF-β signalling (71). Natural products, in parallel, can induce immunogenic cell death through apoptosis, autophagy, pyroptosis and necroptosis, driving dendritic-cell activation and antigen presentation and synergising with immune-checkpoint blockade (71). This mechanism is not unique to any single group: tumour ablation of all modalities—radiofrequency, microwave, cryoablation, irreversible electroporation and high-intensity focused ultrasound—is now understood as a form of *in situ* vaccination, releasing damage-associated molecular patterns such as calreticulin, ATP and HMGB1 that activate cGAS–STING signalling and dendritic-cell maturation (99, 100), and combining ablation with immunoadjuvants can convert immunologically “cold” tumours into “hot” ones and amplify the abscopal effect (101).

**Figure 4:**
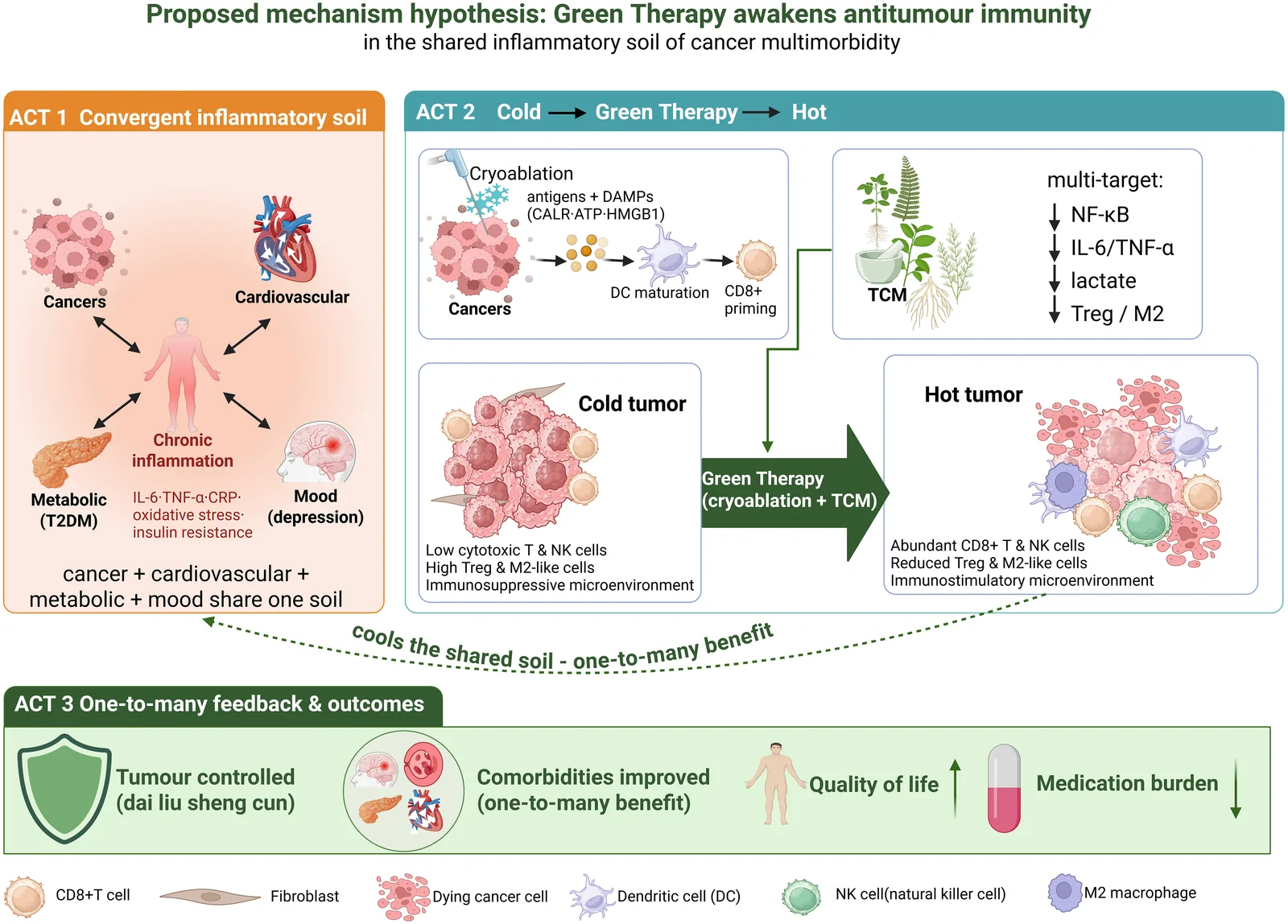
Proposed mechanism hypothesis: cryoablation releases tumour antigens and DAMPs while multi-target TCM dampens inflammation, converging to turn a “cold” tumour microenvironment “hot” and awaken immunity — with hypothesised benefit across the tumour and its comorbidities. Created in BioRender. Li, L. (2026) https://BioRender.com/hz2j73w.

Work from the present group has begun to engineer this synergy explicitly. An *in situ* nanovaccine using Astragalus polysaccharide as immunoadjuvant, combined with cryoablation, captured ablation-released antigens and delivered them to lymph nodes, remodelling the immune microenvironment and producing an abscopal antitumour effect (102). Gallium-based liquid-metal nanoplatforms have been developed as autologous cancer vaccines that destroy orthotopic tumours under external energy stimulation, capture and transport antigens to dendritic cells, expand cytotoxic T lymphocytes and—combined with anti–PD-L1—inhibit abscopal growth, relapse and metastasis (70); a related liquid-metal nanoparticle system releases Ga^3+^ to induce mitochondrial damage and immunogenic cell death, which, combined with irreversible electroporation, mediates durable tumour-specific immunity (103). Together these supply a contemporary scientific interpretation of Green Therapy’s central claim: local minimally invasive destruction and systemic multi-target herbal therapy are not merely additive but *cooperative*, the former generating antigen and the latter remodelling the immunological soil so that the host’s own immunity is awakened (**Figure 4**). Mechanistic reviews from the group extend this to systemic immunometabolism, showing how TCM bioactive compounds—baicalin, berberine, curcumin and icaritin—reprogram tumour lactate metabolism and glycolysis to relieve immune-checkpoint resistance and restore effector T-cell function in hepatocellular carcinoma (89). Related work from the group on programmed cell-death pathways and the tumour immune landscape supplies target-level rationale for syndrome-differentiated intervention (104–106).

### An integrated signalling account

**Figure 5:**
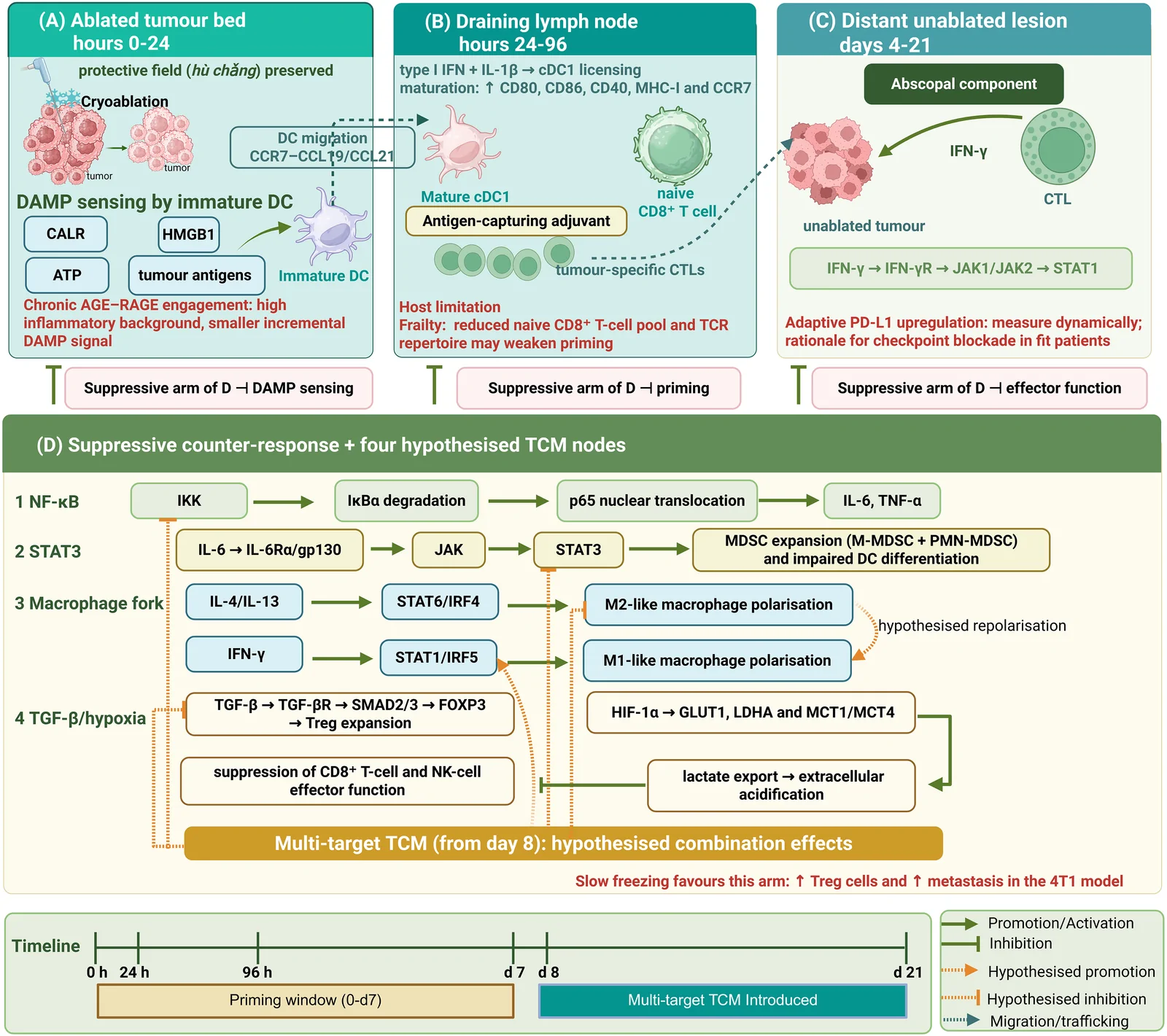
Proposed integrated signalling network. Cryoablation-induced necrosis releases tumour antigen with calreticulin, ATP and HMGB1, which act through CD91, P2RX7–NLRP3–IL-1β, TLR4/RAGE and cGAS–STING–TBK1–IRF3 to license cDC1 cells; licensed dendritic cells migrate via CCR7 to the draining lymph node and cross-prime tumour-specific CD8^+^ T cells, which traffic to ablated and unablated lesions and release interferon-γ, upregulating MHC class I, CXCL9/CXCL10 and, adaptively, PD-L1. In parallel, injury-associated IL-10 and TGF-β, regulatory T-cell expansion, myeloid-derived suppressor-cell recruitment and lactate accumulation constitute a suppressive counter-response. Multi-target TCM is proposed to act on four nodes of that counter-response (NF-κB/IL-6–STAT3; STAT6–IRF4 versus STAT1–IRF5 in macrophages; HIF-1α and monocarboxylate-transporter-dependent lactate export; TGF-β–Foxp3), while the acute signalling of steps 1 and 2 must be preserved—hence the proposed priming window during which strongly anti-inflammatory and antioxidant formulas are withheld. Broken arrows denote hypothesised rather than established interactions in this combination. Created in BioRender. Li, L. (2026) https://BioRender.com/ej0eakw. **(A)** Ablated tumour bed (hours 0–24): DAMP sensing by immature dendritic cells following cryoablation-induced necrosis. **(B)** Draining lymph node (hours 24–96): dendritic-cell licensing, maturation and antigen-capturing adjuvant function priming tumour-specific CD8+ T cells. **(C)** Distant unablated lesion (days 4–21): effector trafficking, expansion and the abscopal immune response. **(D)** The suppressive counter-response and four hypothesised TCM intervention nodes (NF-κB/IL-6–STAT3; macrophage polarisation; HIF-1α/lactate export; TGF-β–Foxp3), shown against a timeline of the priming window and multi-target TCM introduction from day 8 onward.

The account above is mechanistic but has been given at the level of cell populations. We therefore set out the proposed cascade explicitly, in the order in which it is claimed to occur, together with the point at which the systemic arm is claimed to act (**Figure 5**).

#### Step 1 — controlled necrosis and DAMP emission (hours 0–24)

Freeze–thaw injury produces coagulative necrosis with membrane rupture rather than the immunologically silent apoptosis favoured by many cytotoxic drugs. Endoplasmic-reticulum stress drives pre-apoptotic surface exposure of calreticulin, which signals through CD91 on dendritic cells; released ATP signals through P2RX7 to assemble the NLRP3 inflammasome and mature IL-1β; HMGB1 engages TLR4 and RAGE; and released nuclear and mitochondrial DNA taken up by phagocytes activates cGAS–STING and hence TBK1–IRF3 and type I interferon (99, 100).

#### Step 2 — dendritic-cell licensing and antigen transport (hours 24–96)

Type I interferon and IL-1β license conventional type 1 dendritic cells, which upregulate CCR7 and migrate to the draining lymph node, where MHC class I cross-presentation with CD80/CD86 and CD40 costimulation primes tumour-specific CD8^+^ T cells. This is the step at which an antigen-capturing nanovaccine adjuvant intervenes (102).

#### Step 3 — effector expansion, trafficking and interferon-γ feedback (days 4–21)

Primed CD8^+^ T cells expand and traffic both to the ablated bed and to distant unablated deposits, the abscopal component. Interferon-γ released at those sites upregulates MHC class I, induces CXCL9 and CXCL10 to recruit further effectors, and adaptively upregulates PD-L1. That adaptive PD-L1 rise is a mechanistic argument for adding checkpoint blockade in fit patients, and a reason to measure PD-L1 dynamically rather than only at baseline.

#### Step 4 — the suppressive counter-response, and where the systemic arm acts

The same injury drives IL-10 and TGF-β release, regulatory T-cell expansion and recruitment of monocytic and granulocytic myeloid-derived suppressor cells; a slow freeze favours this arm and, in the 4T1 model, increased metastasis (75, 76). Multi-target TCM is proposed to act principally here, on four nodes: NF-κB and IL-6–STAT3 signalling, reducing suppressor-cell expansion and M2 polarisation; the STAT6/IRF4 axis in tumour-associated macrophages, favouring an IRF5/STAT1-dominated phenotype; tumour glycolysis and lactate export through HIF-1α and monocarboxylate transporters, relieving lactate-mediated suppression of effector T and natural killer cells (89); and TGF-β-driven Foxp3 induction. On this account the two modalities act on opposite arms of the same fork—one supplying the stimulatory input, the other removing the suppressive brake—which is why supra-additive rather than merely additive effects are predicted.

A timing consequence follows, and we regard it as one of the more useful outputs of making the cascade explicit. Steps 1 and 2 depend on acute inflammatory signalling—NF-κB, NLRP3–IL-1β and type I interferon—that the systemic arm is expressly designed to dampen. If a strongly anti-inflammatory or antioxidant formula is given during the first days after ablation, the hypothesis predicts that it will blunt rather than amplify priming. The model therefore requires that the two arms be *sequenced*, not merely combined. The potential for outright antagonism is examined under Safety, below, and the timing prediction is embedded as a randomised comparison in the design proposed in **Table 7**.

**Table 7:** Proposed two-stage prospective evaluation of the hypothesis.

Domain	Stage 1: randomised phase II, biomarker-primary	Stage 2: pragmatic phase III, non-inferiority
Design	Open-label, randomised 1:1, multicentre (4–6 sites); embedded second randomisation of herbal start day (day 1 vs day 8)	Open-label, randomised 1:1, pragmatic, multicentre consortium including at least one non-Chinese site
Population	Stage IIIB/IV non-small cell lung cancer	Solid tumours with an ablatable dominant lesion, stratified by tumour type
Key inclusion	Age ≥ 65 (or G8 ≤ 14); ≥ 2 chronic comorbidities; ≥ 5 daily medications; one dominant symptomatic lesion ≤ 5 cm accessible to percutaneous ablation; cold or inflamed-suppressed profile (low CD8 infiltration and/or raised neutrophil-to-lymphocyte ratio, CRP or IL-6); intolerance of, ineligibility for or refusal of full-dose systemic therapy; life expectancy ≥ 3 months	As Stage 1, with eligibility broadened to the tumour types validated in Stage 1
Key exclusion	Fit for curative resection or for standard-dose therapy with proven survival benefit; no dominant lesion; uncorrectable coagulopathy or platelets < 50 × 10^9^/L; lesion abutting critical structures; severe emphysema or contralateral pneumonectomy (lung); checkpoint-inhibitor-eligible with adequate performance status; narrow-therapeutic-index agents with documented major herb–drug interaction	As Stage 1
Intervention	Cryoablation of the dominant lesion with pre-specified rapid freeze–thaw parameters, followed from day 8 by the modular formula (fixed GMP backbone granule + up to 3 pre-specified syndrome-assigned modules), continued 6 months, with protocolised deprescribing review	Stage-adapted Green Therapy per the three-stage model, delivered pragmatically
Comparator	Best supportive care plus standard non-intensive management, with an identical protocolised deprescribing review	Physician’s choice of standard care for this population
Co-primary endpoints	(i) Immunological conversion at week 4: ≥ 2-fold rise in CD8+ density in a distant unablated lesion, or ≥ 2-fold rise in interferon-γ ELISpot to autologous lysate; (ii) change in daily medication count at 6 months	Overall survival (non-inferiority)
Secondary endpoints	Disease control rate; progression-free survival; quality of life (EORTC QLQ-C30); grade ≥ 3 adverse events; comorbidity control (HbA1c, blood pressure, depression score); potentially inappropriate medications; circulating tumour DNA molecular response; hospital days	Quality of life; total medication burden; grade ≥ 3 toxicity; hospital days; cost-effectiveness
Immune monitoring	Tiers 1–3 of **Table 5**; tissue tier in a pre-specified subgroup with a safely accessible second lesion	Tier 1 in all; tiers 2–3 in a nested biomarker cohort
Sample size and assumptions	Conversion 45% vs 15%, two-sided α 0.05, power 80% → 33 per arm; inflated to 40 per arm (n = 80) for attrition and non-evaluable biopsies. The same 40 per arm gives 80% power for a between-arm difference of 1.3 in mean medication change (SD 2.0)	Non-inferiority margin hazard ratio 1.25, one-sided α 0.025, power 80% → approximately 630 deaths; approximately 460 deaths at a margin of 1.30; implying 600–800 patients
Duration and follow-up	24 months’ accrual; 12 months’ minimum follow-up per patient	36 months’ accrual; follow-up to the required number of events, minimum 24 months
Statistical considerations	Gatekeeping procedure for the two co-primary endpoints, specified in the statistical analysis plan; stratification by centre, frailty (G8) and baseline IL-6 tertile; pre-specified interaction test of treatment effect with baseline inflammatory tone (the direct test of P1); pre-specified per-module sensitivity analysis; intention-to-treat primary with per-protocol supportive	Both intention-to-treat and per-protocol required to meet non-inferiority; independent data monitoring committee; interim analysis for futility
Feasibility assessment	Realistic for a single consortium of 4–6 centres	Beyond any single group; requires an international consortium and dedicated funding. Stated explicitly rather than proposing an underpowered substitute

That the broader integrative-oncology community is converging on adjacent priorities lends external support to this direction. The Society for Integrative Oncology and the American Society of Clinical Oncology have jointly issued evidence-based guidelines endorsing integrative modalities—including acupuncture, acupressure, tai chi, qigong and other TCM-adjacent therapies—for cancer-related pain, anxiety and depression, and fatigue (8–10). Green Therapy is designed to complement, not replace, this agenda, extending the same low-harm, whole-patient logic from symptom control to the management of the multimorbid patient as a whole.

## Implementation: western precision, TCM syndrome differentiation and the role of AI

### A division of labour

**Figure 6:**
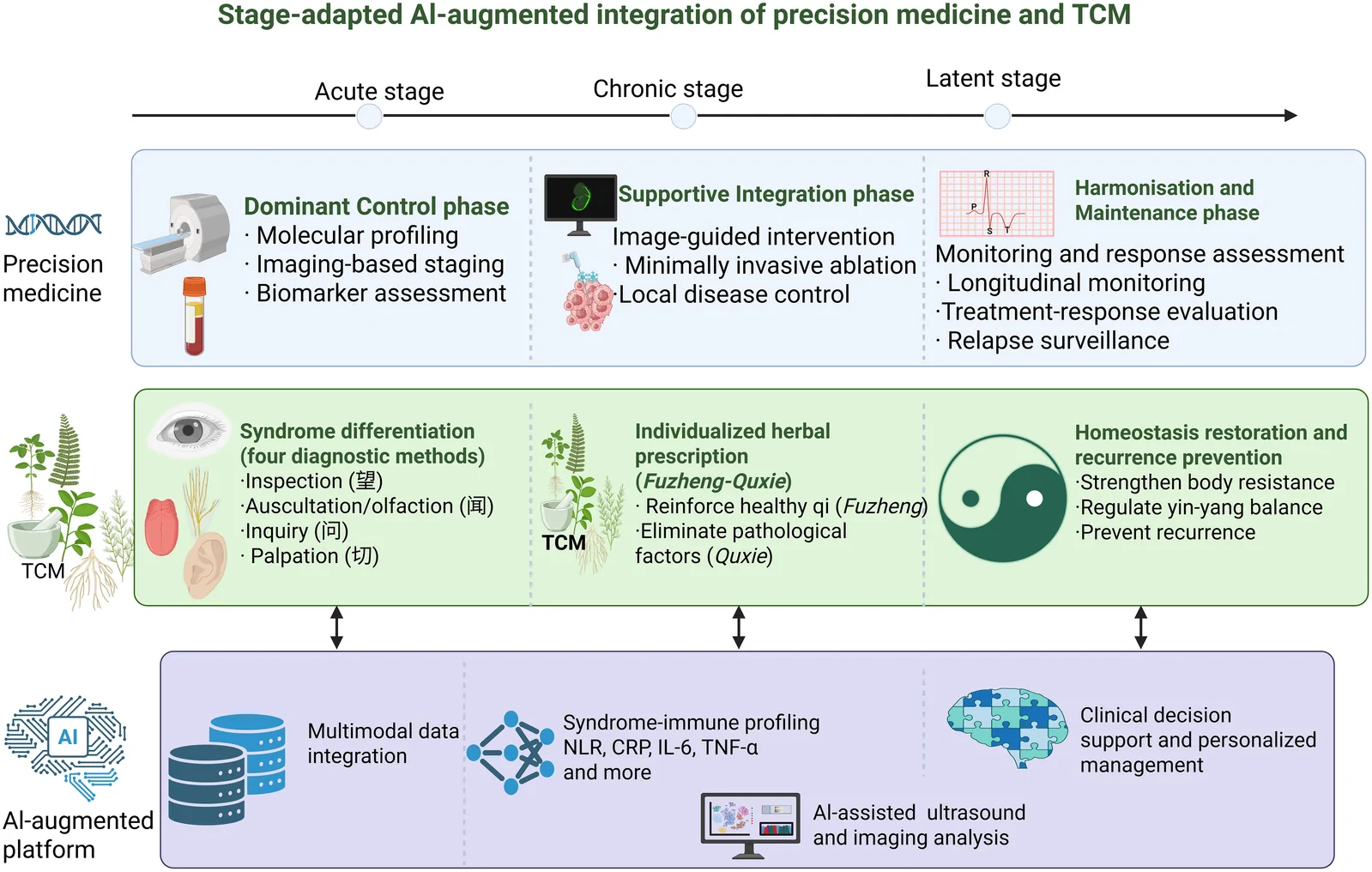
Proposed implementation: Western diagnosis and image-guided ablation paired with TCM syndrome differentiation across the three stages, augmented by AI, with total medication burden as a quantifiable outcome. Created in BioRender. Li, L. (2026) https://BioRender.com/qxegemg.

The implementation model proposed here pairs the complementary strengths of the two medical systems (**Figure 6**). Western medicine contributes molecular and imaging diagnosis, precise tumour staging, genomic and biomarker profiling, and the technical platform for image-guided minimally invasive ablation. TCM contributes syndrome differentiation through the four examinations (inspection, listening-smelling, inquiry and palpation), constitutional assessment, compound herbal prescription calibrated to the patient’s current pathophysiological state, and dynamic readjustment as the disease and its treatment evolve. The two are integrated not as a simultaneous stack but, following the three-stage model, in a stage-dependent, primary/secondary relationship: Western diagnostic precision defines *what* and *where* the disease is and enables decisive local control, while TCM syndrome differentiation defines *who* the patient is and guides sustainable systemic regulation. Deprescribing of redundant disease-specific medications, enabled when a single multi-target formula addresses several comorbid conditions at once, becomes a pre-specifiable, quantifiable indicator of whole-patient benefit.

Operationally, this deprescribing is not *ad hoc*. Candidate medications are identified with validated instruments—the STOPP/START criteria (107) and the AGS Beers criteria (108)—and the classes most often reducible in multimorbid cancer patients (proton-pump inhibitors, long-term benzodiazepines and Z-drugs, redundant antihypertensives, sulfonylureas, and statins of marginal benefit near the end of life) are reassessed at each stage transition. A pharmacist-led pilot in older adults with cancer and polypharmacy identified roughly three times more potentially inappropriate medications using a three-tool screen than with the Beers criteria alone and deprescribed 73% of those identified (109), establishing feasibility; the distinctive contribution of syndrome-differentiated TCM is to make such reductions *sustainable*, because a single multi-target formula can cover several of the indications the discontinued drugs had addressed.

### Patient selection: who is expected to benefit, and who is not

The hypothesis is not a claim about cancer in general, and its clinical value depends on stating the intended population narrowly enough to be tested.

#### Expected to benefit most

Adults, broadly aged 65 years or over (or younger with a G8 score of 14 or less), with a histologically confirmed solid tumour, at least two chronic comorbidities and polypharmacy (five or more daily medications), who have all three of the following: a locally dominant, symptomatic or compressive lesion accessible to percutaneous image-guided ablation and no larger than about 5 cm; an immunologically cold or inflamed-suppressed profile, indicated by low CD8^+^ infiltration and/or elevated neutrophil-to-lymphocyte ratio, CRP or IL-6; and intolerance of, ineligibility for, or informed refusal of standard full-dose systemic therapy. Among tumour types, the local arm is technically established and the systemic evidence least thin in non-small cell lung cancer and hepatocellular carcinoma, and in localised or oligometastatic disease of lung, liver and kidney; our clinical experience is largest in lung cancer, and we would expect any first trial to be conducted there.

#### Where the hypothesis should not be applied

Patients fit for curative resection, or for standard-dose systemic therapy with a demonstrated survival benefit, should receive it. The model is not an alternative to curative treatment, and construing it as one would be the most consequential possible misreading of this article. Patients with high-volume, rapidly progressing disease and no dominant local lesion have little to gain from local debulking, and the absence of a survival benefit from indiscriminate post-chemotherapy cryoablation (66) is the empirical warning. Ablation-specific exclusions apply, including uncorrectable coagulopathy or a platelet count below 50 × 10^9^/L, lesions abutting critical structures, and—for lung ablation—severe emphysema or previous contralateral pneumonectomy. Patients whose tumours are checkpoint-inhibitor eligible on established predictors, with adequate performance status and no contraindicating autoimmune disease, should be considered for checkpoint blockade first or concurrently: this hypothesis addresses the substantial group for whom that option is closed, not those for whom it is open. Finally, patients receiving narrow-therapeutic-index agents for which a major herb–drug interaction is documented—warfarin, CYP3A4-dependent tyrosine-kinase inhibitors, digoxin, tacrolimus—require either substitution of the interacting agent or exclusion.

Stated this way the target population is a definable and clinically substantial minority of incident cancer patients rather than an undifferentiated whole, and these criteria transpose directly into the eligibility criteria of **Table 7**.

### A layered biomarker strategy

The routinely available markers emphasised so far—neutrophil-to-lymphocyte ratio, CRP and IL-6/TNF-α—were chosen for pragmatic reasons: they are inexpensive, obtainable in the county-level hospitals where much of this population is treated, and prognostic in both cancer and cardiometabolic disease, which is precisely the property a one-to-many hypothesis requires. They are nevertheless insufficient to test the mechanistic claim, which concerns antigen-specific immunity. We therefore propose three tiers, specified with their sampling schedule in **Table 5**: a feasibility tier available at every site; a mechanistic tier of central-laboratory immune phenotyping and function, serum DAMPs and type I interferon signature, circulating tumour DNA and T-cell receptor repertoire sequencing; and a tissue tier in a pre-specified subgroup, adding PD-L1 expression, CD8^+^ and CD103^+^ T-cell density, macrophage phenotype, Foxp3^+^ regulatory T cells, an interferon-γ-related gene-expression signature, tumour mutational burden and microsatellite status.

One design point in that tier deserves emphasis, because it is easily got wrong: the paired biopsies must be taken from a distant, *unablated* index lesion, not from the treated lesion, which no longer exists in interpretable form. Only the unablated lesion can demonstrate that systemic, antigen-specific immunity was generated rather than local tissue destruction achieved.

#### Which markers test which claim

This distinction matters more than the list itself. Interferon-γ ELISpot against autologous tumour lysate, T-cell receptor clonal expansion and CD8^+^ density in the unablated lesion test the *in situ* vaccination claim, and therefore P1. Change in IL-6, CRP and neutrophil-to-lymphocyte ratio, together with change in medication count and in objective comorbidity control (glycated haemoglobin, blood pressure, depression score), tests the one-to-many claim, P2. Concordance between AI-derived syndrome scores and these inflammatory markers tests P3. PD-L1 expression, tumour mutational burden and microsatellite status are included chiefly for stratification and as a check on the alternative explanation that any benefit reflects a conventionally immunogenic tumour rather than a modified host. Circulating tumour DNA provides an early, tumour-agnostic efficacy signal in a population in whom post-ablation radiological response is notoriously difficult to interpret. We regard the ELISpot and paired-biopsy components as the elements that convert this from a hypothesis about inflammation into a hypothesis about antitumour immunity, and would fund them before anything else.

## Artificial intelligence as an enabler of syndrome differentiation

The principal practical obstacle to scaling syndrome-differentiated care is that high-quality four-examination diagnosis depends on scarce, experienced physicians and carries inter-observer variability. Artificial intelligence offers a route past this bottleneck, and it is here that the integrative model can be made both more objective and more accessible. Machine-learning models can already identify TCM syndrome types from free-text electronic medical records in cancer patients, a step toward standardised, reproducible syndrome assignment (110). Building on this, the present group and others are developing digital-TCM systems that couple portable, AI-guided ultrasound with large-model–based dynamic evaluation of thyroid and breast nodules, multimodal knowledge-graph platforms for risk-stratifying pulmonary nodules, and augmented-reality “living-inheritance” systems that capture and transmit master physicians’ diagnostic reasoning—several of these supported by national and municipal research programmes. This systems work is ongoing and not yet peer-reviewed, and is described here only to indicate feasibility and direction of travel rather than as validated evidence. Such tools push screening and syndrome assessment toward primary care and the home, lowering the expertise barrier that has historically limited syndrome-differentiated oncology.

A particularly promising direction is the use of AI to construct an objective “immune portrait” from the four examinations. The visible signs read in TCM diagnosis—tongue body and coating, the vascular collaterals of the sclera, and pulse characteristics—plausibly co-vary with systemic inflammatory burden as captured by markers such as the neutrophil-to-lymphocyte ratio, C-reactive protein and IL-6/TNF-α. If AI-quantified four-examination features can be calibrated against such biomarkers, syndrome patterns could be linked to measurable immune-inflammatory states, giving syndrome differentiation a transparent biological correlate and providing a non-invasive readout of the very “soil” that Green Therapy seeks to improve. This proposal is at an early stage and requires prospective validation, but it exemplifies how AI could transform syndrome differentiation from an experiential art into a reproducible, biologically anchored instrument suited to the management of cancer multimorbidity.

### Which computational approaches, and what would count as improvement

“Artificial intelligence” is too broad to be a testable proposal, so we specify the four approaches envisaged, the input each consumes, and the metric by which each should be judged.

#### Computer vision on standardised four-examination inputs

Convolutional and vision-transformer models applied to tongue body and coating, sublingual vein and facial complexion images captured under standardised conditions—fixed working distance, a D65-equivalent illuminant and an in-frame colour calibration target—since acquisition rather than modelling is the dominant source of error in published tongue-image work. The intended output is not a single discrete syndrome label but a continuous probability vector over syndrome elements (qi deficiency, yin deficiency, damp-heat, blood stasis), which is both more faithful to clinical reality and directly usable as a covariate.

#### Radiomics and delta-radiomics

Hand-crafted and deep features from pre-ablation contrast-enhanced CT, used for two distinct purposes: planning the ablation zone and the freeze–thaw protocol, and predicting immunological conversion. Delta-radiomic features from the 24–72-hour and week-4 scans, quantifying the peri-ablational rim, are a plausible non-invasive surrogate for local immune infiltration and would be validated against the tissue tier above.

#### Multimodal fusion

A late-fusion model integrating imaging features, tier 1 and tier 2 laboratory data, four-examination features and structured medication data, with the concrete task of predicting the two co-primary outcomes. Fusion is the point at which the two medical systems become commensurable within a single model rather than merely used side by side.

#### Knowledge graphs and language models

A herb–compound–target–pathway–syndrome–biomarker knowledge graph serves two functions no black-box model can: automated screening for pharmacokinetic and pharmacodynamic herb–drug interactions at the point of prescribing, and attribution of an observed effect to candidate constituents, which is what makes a multi-target formula scientifically tractable. Large language models are envisaged for a deliberately narrow set of tasks—structuring free-text clinical records into syndrome elements (110), extracting herb and dose data from prescriptions, and generating interaction alerts against the knowledge graph—always retrieval-grounded and always with clinician confirmation, and explicitly not for autonomous prescribing, for which their known tendency to generate fluent but unsupported statements is disqualifying.

#### What would count as objective improvement

We propose three pre-specified metrics in place of an assertion of benefit. First, inter-rater reliability of syndrome assignment between independent practitioners, where the target is an increase in Cohen’s κ from the 0.4–0.6 range typically reported to 0.75 or above when both are model-assisted. Second, discrimination of the multimodal model for the co-primary outcomes on prospective external validation at centres contributing no training data. Third, concordance between AI-derived syndrome scores and the tier 1 inflammatory markers, which is the specific test of the “immune portrait” proposal. Reporting would follow TRIPOD+AI and, for prospective evaluation, DECIDE-AI and CONSORT-AI. We state plainly that no system described here, including our own, has yet been evaluated to that standard.

## Safety, herb–drug interactions and the limits of the evidence

A paradigm built on combining herbal formulas with antineoplastic and cardiometabolic drugs must take pharmacological safety as seriously as efficacy, and must be candid about the weaknesses of its own evidence base.

## Herb–drug interactions are real, common and under-reported

The risks are not hypothetical. St John’s Wort, a potent inducer of CYP3A4 and P-glycoprotein, reduces exposure to the active irinotecan metabolite SN-38 by 42% and lowers imatinib area-under-the-curve by 30–43%—enough to compromise efficacy (111–114); the same enzyme system underlies the interaction between grapefruit and oral tyrosine-kinase inhibitors such as nilotinib (115). Pharmacodynamic interactions are equally consequential: American ginseng blunts the anticoagulant effect of warfarin (116), whereas *Salvia miltiorrhiza* (danshen) potentiates it and has caused gross over-anticoagulation (117, 118). The last example carries a pointed clinical irony for this review—danshen is a blood-activating herb indicated for exactly the blood-stasis, hypercoagulable patients who are most likely to be receiving anticoagulation for cancer-associated thrombosis. These hazards are magnified by scale and by silence: among breast and prostate cancer patients, 84% used herbs or supplements and 38% of the resulting potential interactions were classified as major (119), yet between a fifth and three-quarters of patients never disclose such use to their oncologist (120). A living, mandatory herb–drug interaction registry and routine medication reconciliation are therefore non-negotiable components of the model, not optional refinements.

### Where the two arms could work against each other

The hypothesis as stated is one of synergy, and a balanced account must specify the conditions under which the same two modalities would be expected to antagonise each other. Four are mechanistically plausible; all four are addressable by design, which is why we regard them as constraints on the protocol rather than objections to the model.

#### Blunting of immunogenic cell death

Calreticulin exposure and the wider DAMP programme depend on reactive-oxygen-species-driven endoplasmic-reticulum stress, and dendritic-cell licensing depends on NLRP3–IL-1β and type I interferon signalling. Formulas rich in antioxidant constituents, and heat-clearing (*qing re*) formulas selected precisely for NF-κB-inhibitory activity, could therefore attenuate the very priming event that ablation is intended to generate (121, 122). The model accordingly specifies a priming window—from the procedure to approximately day 7—during which high-dose antioxidant and strongly anti-inflammatory formulas are withheld, after which the immunoregulatory arm is introduced. This is falsifiable rather than precautionary: a trial randomising the herbal arm to begin on day 1 versus day 8 tests it directly, and we propose exactly that in **Table 7**.

#### Haemorrhagic risk from blood-activating herbs

Blood-activating, stasis-resolving (*huo xue hua yu*) herbs—*Salvia miltiorrhiza*, *Ligusticum chuanxiong*, *Panax notoginseng*, *Carthamus tinctorius*—have antiplatelet and fibrinolytic activity, and danshen potentiates warfarin (117, 118). These are indicated for exactly the blood-stasis pattern that maps onto the hypercoagulable axis, and therefore for exactly the patients receiving anticoagulation for cancer-associated thrombosis, while percutaneous cryoablation of lung or liver carries a real haemorrhagic risk. The pattern-indicated herb and the procedural risk point in opposite directions. The model requires that these herbs be withheld for at least five to seven days before ablation and resumed only after imaging has excluded significant haemorrhage.

#### Impaired healing of the ablation cavity

Constituents with antiangiogenic or antiproliferative activity could plausibly delay organisation of the cryolesion, with cavitation, delayed pneumothorax and bronchopleural fistula the clinically relevant concerns in lung ablation. Direct evidence is sparse and we flag this as an acknowledged gap rather than an established effect; the model therefore treats the first two peri-procedural weeks as a period for tonifying rather than attacking (*gong xie*) strategies.

#### Frank immunosuppression

Some traditional agents used in oncology and rheumatology, notably *Tripterygium wilfordii*, have well-characterised immunosuppressive activity (123–125). Whatever their value in other indications, they are mechanistically incompatible with a strategy whose object is T-cell priming, and the model treats them as contraindicated in the peri-ablation period. Herb-induced liver injury belongs here as a related hazard: it is not itself an antagonism, but it confounds attribution of hepatotoxicity in any patient concurrently receiving antineoplastic agents, and it argues for scheduled liver-function monitoring and for not introducing a new formula and a new systemic agent in the same week.

Two further caveats belong here. Immunostimulatory polysaccharides, including the Astragalus polysaccharide used as an adjuvant in our own work (102), have dose-dependent and in some settings biphasic effects on myeloid populations, and a dose that expands suppressor rather than antigen-presenting populations cannot be excluded *a priori*; dose-finding is a prerequisite, not a refinement. And the “cold” character attributed to cryoablation within the classical framework, combined with cooling (*han liang*) herbs, would in traditional terms risk injuring spleen and stomach yang in an already deficient patient—a prediction that, unlike most syndromic claims, is straightforwardly testable through gastrointestinal adverse-event rates.

## Product quality and the cautionary lesson of aristolochic acid

The agents themselves are heterogeneous. One-fifth of surveyed herbal products have contained detectable lead, mercury or arsenic (126), and batch-to-batch variability in bioactive content remains a real obstacle to reproducibility. The gravest lesson is aristolochic acid: products adulterated with *Aristolochia* caused an epidemic of nephropathy and urothelial carcinoma—46% of one cohort developed urothelial cancer (127)—and the carcinogen leaves an unmistakable genome-wide mutational signature now detected in urothelial, renal and hepatocellular cancers (128–130). That a traditional herb can itself cause cancer is the strongest possible argument that “natural” is not “safe,” and that Green Therapy must rest on pharmacovigilance, botanical authentication and standardisation rather than on tradition alone.

### From variability to a trial-grade product: standardisation, quality control and regulatory path

Acknowledging heterogeneity is not the same as solving it, and a multicentre trial cannot be run on an unspecified product. What follows is the minimum specification we consider necessary, and it is simultaneously an admission of how far current practice is from it.

#### Botanical identity and chemical consistency

Species authentication by macroscopic and microscopic examination together with DNA barcoding (ITS2 and psbA-trnH), which resolves the substitution and adulteration that morphological inspection alone does not; chromatographic fingerprinting by HPLC-DAD and UPLC-Q-TOF-MS against a pre-registered reference fingerprint with a declared similarity acceptance threshold; quantitative assay of at least three marker constituents per herb, chosen for pharmacological relevance rather than analytical convenience, with pre-set acceptance ranges; and content-uniformity and dissolution testing for granule products.

#### Safety specifications

Heavy metals, pesticide residues, aflatoxins, sulphur dioxide and microbial limits tested against the current Chinese Pharmacopoeia and, for a multiregional trial, against European Pharmacopoeia and USP limits; and explicit exclusion of, and analytical screening for, aristolochic-acid-containing taxa, given the history described above.

#### Manufacturing discipline

Manufacture of the entire trial supply under GMP from a single documented batch of each herb wherever quantity permits, with retention samples, stability data covering the trial duration, and certificates of analysis released to the trial statistician. Blinding requires a placebo granule matched for appearance, odour and taste, which for aromatic preparations is genuinely difficult and is one substantive reason double-blind TCM trials remain rare; where blinding is unattainable, blinded outcome assessment and objective endpoints must carry the burden.

#### Reconciling individualisation with standardization

This is the real tension, and it cannot be dissolved by asserting that both matter. A fully individualised prescription is unstandardisable; a fixed formula abandons syndrome differentiation, which is the systemic arm’s proposed mechanism of patient-matching. We therefore propose a modular design: a fixed, GMP-manufactured backbone granule given to every patient in the experimental arm, plus up to three pre-specified modifier modules, each separately manufactured and quality-controlled, each with defined syndrome-based indications and doses, assigned by protocol algorithm and recorded per patient. This preserves a reproducible core, keeps the number of distinct products small enough to control, permits pre-specified per-module sensitivity analyses, and converts individualisation from a source of uncontrolled variance into an auditable trial variable. We regard this as the most important practical concession the paradigm must make in order to be testable.

#### Regulatory path

In China the route depends on classification—hospital preparation, classic-formula catalogue product, or new TCM drug—and multicentre interventional use requires the corresponding national pathway together with ethics approval at each site. For any trial intended to support use outside China, the United States botanical-drug framework, under which PHY906 has been developed (93–95), and the European herbal-monograph system define the applicable expectations, chiefly around characterisation and batch consistency. We regard cross-jurisdictional acceptability as an unsolved problem for compound formulas at present, and one that a modular, chemically fingerprinted product is considerably more likely to survive than a bespoke decoction.

## The evidence base has structural weaknesses

Finally, the clinical literature must be read critically. Trials of TCM have historically suffered from inadequate randomisation and blinding, small samples and short follow-up (131), and reporting is distorted by publication bias so pronounced that nearly all trials from some regions report positive results (132). Network-pharmacology analyses, for all their mechanistic appeal, are predominantly predictive and require experimental and clinical confirmation. None of this refutes the Green Therapy hypothesis, but it sets the evidentiary bar: the paradigm’s central claim—that it can reduce medication burden without compromising oncological control—must be proven in adequately powered, transparently reported pragmatic trials, to which we now turn.

### Publication bias, heterogeneity, geography and the translation gap

Four weaknesses deserve more than acknowledgement, because each bounds what the present evidence can support.

#### Publication bias

The distortion here is not marginal. In the systematic review cited above, trials from certain regions reported positive results at rates approaching unity, which is not a credible distribution of true effects (132); the problem is compounded by publication in journals not indexed internationally, by trial registration that is recent and incomplete, and by outcome reporting often selected after the fact. We therefore made a deliberate methodological choice not to pool the TCM efficacy literature quantitatively, because meta-analysis of a literature with a near-unity positive rate mainly propagates the bias with narrower confidence intervals. The aggregate impression conveyed by the efficacy citations in this article should be read as an upper bound.

#### Heterogeneity of the intervention itself

“TCM” is not an intervention. Huaier granule, PHY906, Shenqi Fuzheng injection and a physician’s individualised decoction share a tradition and almost nothing pharmacologically; a positive result with one carries little evidential weight for another. This is more serious than batch variability within a product, because it means the field’s apparent evidence base is an aggregate over non-exchangeable interventions. Our own argument is exposed to this: the pivotal trial evidence cited above (92–97) supports specific products, not the paradigm.

#### Geographic and design concentration

Almost all clinical data on cryoablation combined with TCM originate from Chinese centres and are largely retrospective or single-arm, with cryoablation technique, device, freeze–thaw protocol and concomitant care varying between them and differing from practice elsewhere. There has been no independent replication outside China of the specific combination proposed here, so external validity is unestablished. We consider independent replication—ideally including a non-Chinese centre in any multicentre trial—more informative than a further domestic single-arm series.

#### The preclinical-to-clinical gap

The mechanistic work on which the immunological argument rests, including our own, uses young, lean, immunocompetent mice bearing a single transplanted tumour. The hypothesis concerns aged, multimorbid, chronically inflamed hosts whose immune systems differ in precisely the respects the hypothesis invokes. This is not a generic caveat but a specific mismatch between model and claim, and it identifies the preclinical priority named earlier: replication in aged, diet-induced-obese and diabetic tumour-bearing animals before, not after, a large clinical trial. The frequency with which local and immunological responses to ablation have failed to translate into survival benefit (66) should be read as evidence that this gap is real rather than as a technicality.

## Future directions and research priorities

The hypothesis that Green Therapy can deliver durable, low-harm control of cancer multimorbidity requires systematic empirical testing before it can be considered established. Five priorities follow directly. First, pragmatic randomised trials are needed in which patients with cancer and at least two comorbid conditions are randomised to standard care versus stage-adapted Green Therapy, with overall survival, quality of life and total medication burden (deprescribing) as pre-specified endpoints; existing cryoablation trials indicate that careful patient selection will be decisive (66, 74). Second, standardisation of syndrome differentiation in oncology must be validated, with inter-rater reliability studies and AI-assisted pattern-recognition tools enabling syndrome-differentiated prescription to be applied reproducibly across centres (110). Third, pharmacovigilance for herb–drug interactions should be built and maintained as a living, evidence-based database, since combining compound formulas with antineoplastic and cardiometabolic agents carries real interaction risk. Fourth, biomarker development linking syndrome patterns to molecular and immune phenotypes—including the AI-derived immune portrait described above—would strengthen the mechanistic basis of the paradigm and allow patient selection to be refined. Fifth, health-economic modelling is required to quantify cost-effectiveness; if stage-adapted Green Therapy reduces polypharmacy and avoids the costs of radical treatment and its complications in frail patients, the downstream savings could be substantial, but this must be demonstrated rather than assumed.

### A concrete prospective test, and what would refute the hypothesis

Scrutiny of a hypothesis paper properly asks not only what evidence would support the hypothesis but what would defeat it. We therefore set out a two-stage prospective programme (**Table 7**) and pre-specify the observations we would accept as refuting each proposition (**Table 2**).

Stage 1 is a randomised, open-label, biomarker-primary phase II trial in stage IIIB/IV non-small cell lung cancer in patients meeting the selection criteria above, comparing stage-adapted Green Therapy—cryoablation of a dominant lesion followed by the modular formula from day 8—with best supportive care plus standard non-intensive management. The co-primary endpoints are immunological conversion (a doubling of CD8^+^ density in a distant unablated lesion at week 4, or a doubling of the interferon-γ ELISpot response to autologous tumour lysate) and change in daily medication count at six months. Assuming conversion in 45% versus 15%, with two-sided α of 0.05 and 80% power, 33 patients per arm are required, rising to 40 per arm to allow for attrition and non-evaluable biopsies; the same 40 per arm gives 80% power to detect a between-arm difference of 1.3 in mean medication change, assuming a standard deviation of 2.0. Eighty patients across four to six centres over 24 months is realistic, and the two co-primary endpoints would be tested under a gatekeeping procedure specified in the statistical analysis plan.

Stage 2, contingent on Stage 1, is a pragmatic open-label non-inferiority trial with overall survival as the primary endpoint. We state the arithmetic plainly, because it is the main obstacle: with a non-inferiority margin of hazard ratio 1.25, one-sided α of 0.025 and 80% power, approximately 630 deaths are required, falling to roughly 460 at a margin of 1.30—implying 600 to 800 patients and a multicentre consortium that should, in our view, include at least one non-Chinese site. A trial of this size is beyond any single group, and saying so is more useful than proposing an underpowered substitute. The registered protocol comparing Green Therapy with a platinum doublet (74) is a step towards it but is not powered for the treatment-burden claim.

#### Timing as a randomised variable

Because the signalling account above makes timing a mechanistic prediction rather than a convention, we propose that Stage 1 embed a second randomisation of the herbal start day (day 1 versus day 8 after ablation). The priming-window argument predicts that early initiation of anti-inflammatory formulas will reduce immunological conversion; a null result would falsify one of the more specific claims made here, at modest incremental cost.

Three general failure modes would count against the hypothesis as a whole even if individual endpoints were met: immunological conversion unrelated to baseline inflammatory tone, which contradicts P1; medication reduction achieved at the cost of deteriorating comorbidity control, which satisfies P2 in form while contradicting it in substance; and syndrome assignments that fail to correlate with any inflammatory or immune measure, which contradicts P3. We would regard each as a genuine refutation rather than an occasion for reformulation.

### Alternative explanations we cannot yet exclude

Balance requires naming the explanations that would account for the existing clinical observations without this hypothesis being true. Three are live. The first is selection: patients offered cryoablation with TCM in retrospective series are those well enough to attend repeatedly, and the 19-month median survival reported in such a series (72) is compatible with selection alone. The second is that the benefit of the combination is attributable entirely to the local arm, with the herbal arm contributing supportive-care effects on appetite, sleep and mood that improve tolerance and adherence without any immunological action; this would leave the clinical practice defensible and the mechanistic hypothesis false, and only the antigen-specific endpoints of tier 2 can separate the two. The third is that any reduction in medication count reflects the deprescribing consultation and closer follow-up rather than pharmacological substitution by the formula—in our judgement the most probable confounder of the one-to-many claim, which is why the design above applies an identical deprescribing review to both arms. Stating these alternatives is not a rhetorical concession: the second and third are the reason the trial in **Table 7** is designed as it is.

## Conclusions

Cancer is increasingly a chronic disease of older, multimorbid patients in whom the tumour is only one of several simultaneous problems. The dominant single-disease, subspecialised model, for all its precision, structurally generates additive prescribing cascades and treatment burden that this population tolerates poorly, and it offers no unified framework for the shared pathophysiological soil from which coexisting diseases grow. Tumour Green Therapy, proposed by Hu and colleagues, offers such a framework: a low-harm, quality-of-life–centred paradigm that, through a three-stage model and two flexibly combined modalities—minimally invasive cryoablation and Traditional Chinese Medicine—seeks to control rather than eradicate the tumour while awakening the patient’s own immunity. Its biological plausibility is supported by converging evidence on shared disease mechanisms, by the multi-component, multi-target pharmacology of TCM compound formulas validated in pivotal trials, and by mechanistic work showing that ablation and herbal therapy cooperate to drive immunogenic cell death and remodel the immune microenvironment. Paired with Western diagnostic precision and increasingly enabled by artificial intelligence, syndrome-differentiated Green Therapy is a coherent and testable strategy for cancer multimorbidity. We advance it as a hypothesis-generating paradigm whose promise must now be confirmed in pragmatic trials with survival, quality of life and treatment burden as the measures that matter.

## Data Availability

The original contributions presented in the study are included in the article/supplementary material. Further inquiries can be directed to the corresponding authors.
